# Bioinformatics Analysis of Metabolism Pathways of Archaeal Energy Reserves

**DOI:** 10.1038/s41598-018-37768-0

**Published:** 2019-01-31

**Authors:** Liang Wang, Qinghua Liu, Xiang Wu, Yue Huang, Michael J. Wise, Zhanzhong Liu, Wei Wang, Junfeng Hu, Chunying Wang

**Affiliations:** 1 Department of Bioinformatics, School of Medical Informatics, Xuzhou Medical University, Xuzhou, Jiangsu China; 2 Jiangsu Key Laboratory of New Drug Research and Clinical Pharmacy, School of Pharmacy, Xuzhou Medical University, Xuzhou, Jiangsu China; 3 The Marshall Centre for Infectious Diseases Research and Training, University of Western Australia, Perth, Western Australia Australia; 4 Department of Computer Science and Software Engineering, School of Physics, Mathematics and Computing, University of Western Australia, Perth, Western Australia Australia; 5 Xuzhou Infectious Diseases Hospital, Xuzhou, Jiangsu China; 6 School of Public Health, Capital Medical University, Beijing, China; 7 School of Medical Sciences, Edith Cowan University, Perth, WA Australia; 8 Department of Computer Science, School of Medical Informatics, Xuzhou Medical University, Xuzhou, Jiangsu China

## Abstract

Energy storage compounds play crucial roles in prokaryotic physiology. Five chemical compounds have been identified in prokaryotes as energy reserves: polyphosphate (polyP), polyhydroxyalkanoates (PHAs), glycogen, wax ester (WE) and triacylglycerol (TAG). Currently, no systematic study of archaeal energy storage metabolism exists. In this study, we collected 427 archaeal reference sequences from UniProt database. A thorough pathway screening of energy reserves led to an overview of distribution patterns of energy metabolism in archaea. We also explored how energy metabolism might have impact on archaeal extremophilic phenotypes. Based on the systematic analyses of archaeal proteomes, we confirmed that metabolism pathways of polyP, PHAs and glycogen are present in archaea, but TAG and WE are completely absent. It was also confirmed that PHAs are tightly related to halophilic archaea with larger proteome size and higher GC contents, while polyP is mainly present in methanogens. In sum, this study systematically investigates energy storage metabolism in archaea and provides a clear correlation between energy metabolism and the ability to survive in extreme environments. With more genomic editing tools developed for archaea and molecular mechanisms unravelled for energy storage metabolisms (ESMs), there will be a better understanding of the unique lifestyle of archaea in extreme environments.

## Introduction

Energy storage compounds are important for prokaryotic physiological activities such as regulatory signalling, intracellular persistence, pathogenicity and environmental long-term survival^1^. Wilkinson^2^ first proposed three pre-requisites for defining an energy storage compound, according to which, five energy storage compounds have been confirmed to exist, that is, polyphosphate (polyP), glycogen, and storage lipids such as polyhydroxyalkanoates (PHAs), triacylglycerol (TAG), and wax ester (WE)^3^. These are considered as energy storage mechanisms (ESMs)^1^. Distribution patterns of metabolism pathways of the five energy storage compounds were previously investigated in bacteria^1^. However, currently there is no study focusing on how pathways of energy storage compounds are distributed in archaea. Considering that many archaea are extremophiles, theoretical study of ESMs via pathway distributions would be beneficial to dissect archaeal extremophilic physiology, hence a better understanding of life’s adaptations to extreme environments^4^. An introduction to the five energy storage compounds in prokaryotes is briefly summarized below.

## Polyphosphate

**Figure 1:**
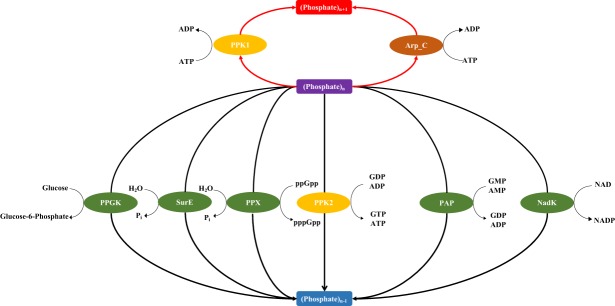
Illustration of polyP metabolism and pathway distributions in archaea. **(a)** Red arrows represent synthesis pathways (PPK1 and Arp_C) that convert (phosphate)_n_ to (phosphate)_n+1_ while deep blue arrows represent independent degradation pathways (PPGK, SurE, PPX, PAP, PPK2 and NadK) that break down (phosphate)_n_ into (phosphate)_n-1_. Arp_C is an abbreviation of actin protein complex of ArpABCEFGH that shares the same Pfam domain actin (PF00022)^8^. **(b**) Eight polyP metabolism pathways and nine archaeal physiology types are presented. Full_Path (7 archaea) encompasses polyP-related six enzymes while No_Path (64 archaea) have lost all six enzymes from archaeal proteomes. Functional_Path (99 archaea) means archaea having PPK1 and at least one polyP-degrading enzyme. PPK1_PPK2 (18 archaea), PPK1_PAP (30 archaea), PPK1_SurE (88 archaea), PPK1_PPX (39 archaea), and PPK2_NADK (78 archaea) means archaea having PPK1 and the designated polyP-degrading enzyme. For each pathway, average proteome size (APS) and GC content were given at the bottom of each boxplot.

Although it is known that polyP is ubiquitous from bacteria to mammals, its metabolism pathway has not been fully elucidated so far and its accumulation is only identified sporadically in archaea through experimental study^5–7^. Beyond its roles as a phosphorus resource and energy reserve, polyP is currently also linked to a set of physiological functions in extremophilic archaea, such as metal resistance, salt tolerance, oxidative stress adaptation, temperature tolerance, and other environmental stresses^6^. Thus, polyP plays important roles for archaeal physiological adaption to environmental changes and stress conditions where archaea reside. In bacteria, enzymes directly linked with polyP metabolism include polyphosphate kinase (PPK1), polyphosphate kinase 2 (PPK2), polyphosphate:AMP phosphotransferase (PAP), 5′/3′-Nucleotidase (SurE), exopolyphosphatase (PPX), NAD kinase and polyphosphate glucokinase^8^. Detailed functions for each enzyme is listed in a previous study by Wang *et al*.^8^ A recent phylogenetic study revealed that PPK1 and PPK2 are less common in bacteria and other unknown enzymes may involve in polyP metabolism^7^. In addition, evolutionary analysis showed that PPK2 evolves earlier than PPK1, matching with Arthur Kornberg’s theory that polyP utilization is much older than synthesis^7^. There is also an actin-related protein complex encoded by *arpABCEFGH* that may fulfil the role of the unknown mechanism for polyP synthesis in bacteria^9^. Although it was proposed that enzymes involved in polyP metabolism show structure conservation among bacteria and archaea, only two enzymes PPX and PPK were analysed in archaea through comparative genomics and there is no overview of a complete polyP metabolism in archaea (Fig. 1)^6^.

## Glycogen

**Figure 2:**
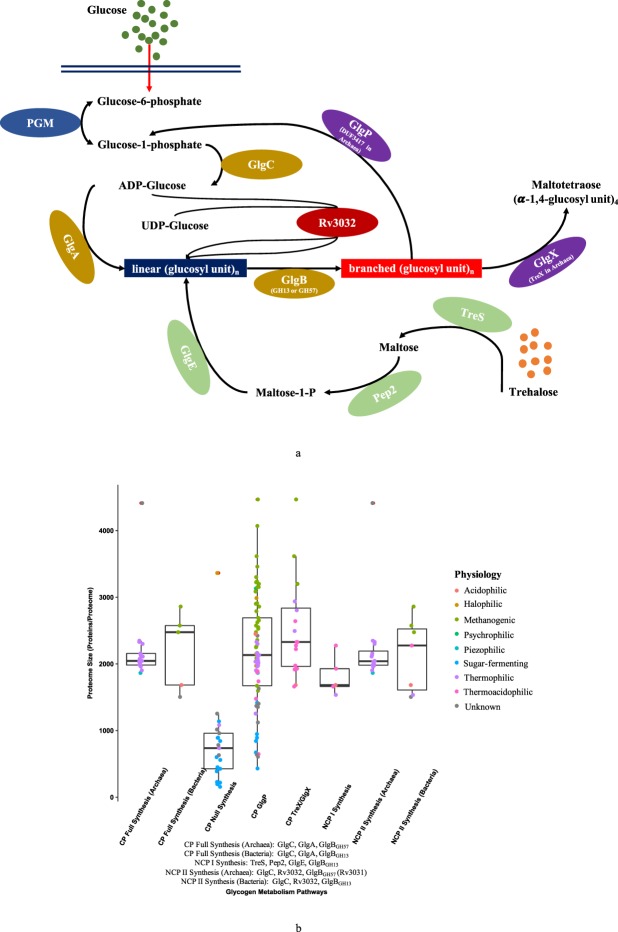
Illustration of glycogen metabolism and pathway distributions in archaea. **(a)** Three pathways are present: (1) classical pathway (CP) of glycogen metabolism (synthesis enzymes GlgC, GlgA and GlgB in dark yellow and degradation enzymes GlgP and GlgX in purple) that is widely distributed in bacterial species; (2) non-classical pathway I (NCP I) including TreS, Pep2, GlgE and GlgB, which links trehalose and maltose metabolism with glycogen; (3) non-classical pathway II (NCP II) including GlgC, Rv3032, and GlgB, which is responsible for glycogen formation and synthesis of 6-O-methylglucosyl-containing lipopolysaccharides (MGLP), a pathogenic factor. PGM is phosphoglucomutase transforming glucose-6-phosphote into glucose-1-phosphate for glycogenesis. **(b)** Distribution of glycogen metabolism pathways in 427 archaeal species. Eight groups of enzymes are formulated, which are CP Full Synthesis (Archaea), CP Full Synthesis (Bacteria), CP Null Synthesis, CP GlgP, CP TreX/GlgX, NCP I Synthesis, NCP II Synthesis (Archaea), NCP II Synthesis (Bacteria). Enzymes in five major groups were detailed at the bottom of the figure.

Carbohydrate polymers and oligomers are stored by living cells for a variety of purposes, such as energy storage and stress resistance. Glycogen is one of the most widely spread carbohydrates that have been identified in archaea, bacteria and eukaryotes^10^. The classical pathway (CP) of bacterial glycogen metabolism includes five essential enzymes that are ADP-glucose pyrophosphorylase (GlgC), glycogen synthase (GlgA), glycogen branching enzyme (GlgB), glycogen phosphorylase (GlgP), and glycogen debranching enzyme (GlgX)^1^. A second glycogen synthesis pathway (non-classical pathway I, abbreviated as NCP I) was recently identified. It involves four enzymes TreS, Pep2, GlgE and GlgB, which correlates trehalose metabolism with glycogen metabolism^11^. Another Rv3032-centric pathway (non-classical pathway II, abbreviated as NCP II) is responsible for lipopolysaccharide and putative glycogen biosynthesis in *Mycobacteria* spp^12^. A brief summary of glycogen metabolism pathways is illustrated in Fig. 2. Previous analysis showed that most archaeal species lack the genes for the classical glycogen pathway, and there is only one archaeal species *Picrophilus torridus* DSM 9790 with all four enzymes in NCP I pathway through BLAST search^11^.

## Storage Lipids

**Figure 3:**
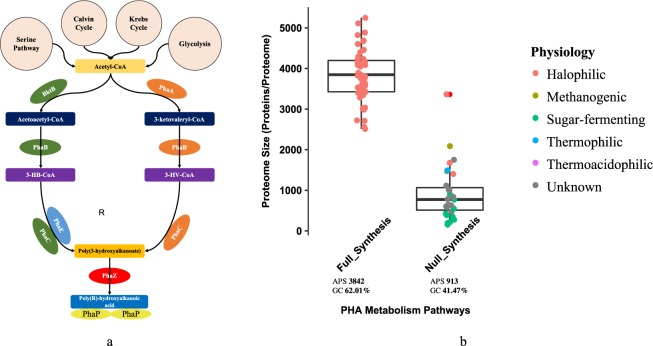
Illustration of classical PHAs metabolism and pathway distributions. **(a)** There are currently four types of PHA synthase system identified. Type I, III, and IV are related with SCL PHAs. Type I has a single PhaC, Type III has a complex of PhaC and PhaE while Type IV is recently identified that consists of PhaC and PhaR. In this study, Type I and Type III synthase systems are considered. The full metabolism pathway includes enzymes PhaA, PhaB, PhaC, PhaE, PhaP, and PhaZ. Since BktB and PhaA share the same protein domain organizations (PF00108 and PF02803) that consist of Thiolase-N and Thiolase-C, only PhaA was studied in this study in order to avoid redundancy. **(b)** Distribution of PHA metabolism pathways in 427 archaeal species. Only synthesis pathways with complete and null enzymes are analysed. Full_Synthesis (51 archaea) group includes PhaA, PhaB, PhaC, PhaE, and PhaP. No_Synthesis (31) group does not have any of these enzymes. For each pathway, average proteome size (APS) and GC content were given at the bottom of each boxplot.

**Figure 4:**
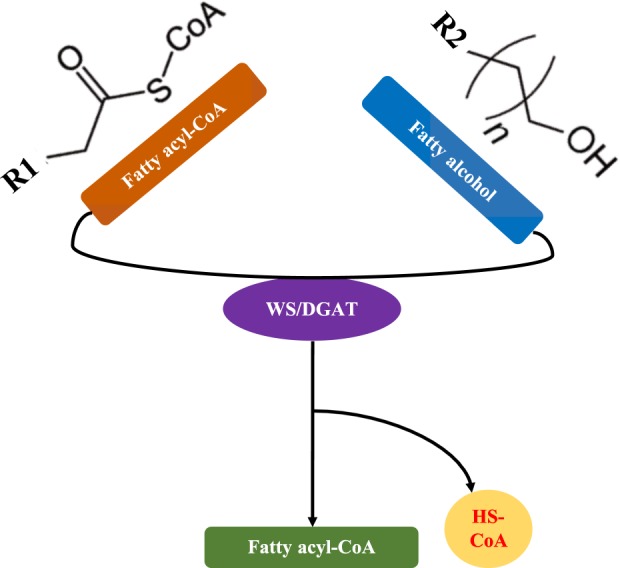
Illustration of wax ester synthesis pathway in prokaryotes. The bifunctional enzyme wax ester synthase/acyl-coenzyme A: diacylglycerol acyltransferase (WS/DGAT) that is responsible for the synthesis of both TAG and WE, is emphasized in this study.

**Figure 5:**
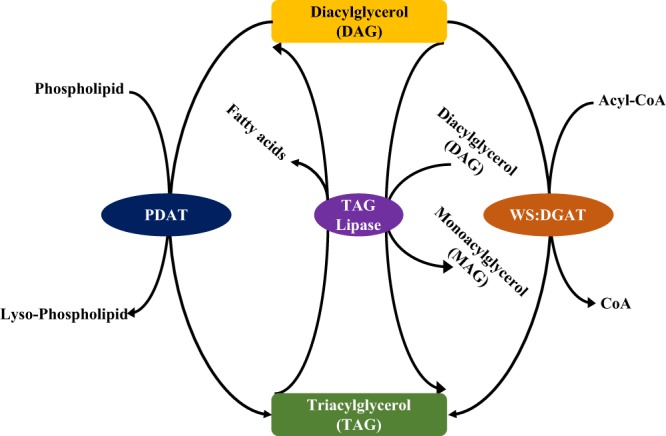
Illustration of TAG metabolism pathway. Phospholipid:diacylglycerol acyltransferase (PDAT) is only present in yeast and plants. Thus, it is not considered in this study. Triacylglycerol lipase is sourced from *Pseudomonas aeruginosa* that reversibly converts Triacylglycerol and Diacylglycerol. WS/DGAT is the same enzyme as in WE synthesis.

There are three common storage lipids in bacteria and archaea: polyhydroxyalkanoates (PHAs) (Fig. 3), wax ester (WE) (Fig. 4), and triacylglycerol (TAG) (Fig. 5). PHAs are a group of naturally-occurring bio-polyesters accumulating in prokaryotes as major energy and carbon sources. They are composed of (*R*)-hydroxy fatty acids and are classified into three categories according to the number of units: short chain length (SCL) PHA, medium chain length (MCL) PHA and long chain length (LCL) PHA. According to the functional alkyl *R* group, PHAs are assigned with specific names and carbon numbers, such as poly-β-hydroxybutyrate (C4) and poly-β-hydroxyvalerate (C5)^13^. Among the family of PHAs, the best known is poly-β-hydroxybutyrate (PHB). Recent studies supported that archaea mainly store PHB and PHV intracellularly^14^. In addition, a mixed PHA consisting of PHB and PHV is also reported in archaea^15^. As for chain length, archaea normally accumulate SCL PHA^16^. A variety of studies confirmed that PHA metabolism involves PhaA, PhaB and PhaC in the synthesis pathway and PhaZ in the degradation pathway (Fig. 3). In addition, synthase subunit PhaE and PHA granule associated protein PhaP are also a part of the synthesis mechanism^14,17^. A novel bifunctional enzyme wax ester synthase/acyl-CoA: diacylglycerol acyltransferase (WS/DGAT) discovered initially in *Acinetobacter calcoaceticus* ADP1 was shown to be present in numerous Gram-positive and Gram-negative bacteria, hence confirmation of wide spread distribution of WE and TAG storage lipids in bacteria^18,19^. It was previously proposed that lipid droplets in archaea accumulated PHA exclusively, while the other two storage lipids, wax ester and triacylglycerol, only arose in bacterial lineages^4^. Initial BLAST search found out 18 homologous WS/DGAT enzymes in archaeal genomes^18^. However, a subsequent analysis only saw a WS/DGAT homologue in one archaeon, which raised the question about how WS/DGAT is distributed in archaea^19^.

In this study, we collected as seed sequences all the enzymes directly linked with metabolism of five energy storage compounds in archaea. Hidden Markov models of all seed proteins were constructed *ab initio* by following standard procedures^3,8^. A total of 427 archaeal reference proteomes were analysed for pathway distribution. A variety of correlational analyses in terms of pathway distribution patterns, proteome size, and archaeal physiology were performed. This study is the first comparative genomic study of energy storage compounds in archaea. The significance of the study resides in identification of the linkages between energy storage compounds and archaeal extremophilic phenotypes, which will promote our understanding of archaeal adaptation to extreme environments. Finally, a set of archaeal enzymes involving ESMs are also discovered, which may have potential benefits for industrial and clinical applications in future studies.

## Results and Discussion

HMM-based screening of archaeal proteomes based on selected seed enzymes that are directly linked to ESMs generated a complete map of pathway distributions (Supplementary Table S1). In addition, literature and database mining (UniProt^20^ and PATRIC^21^) classified 427 archaeal species into three predominant sub-categories, that is, halophiles, thermophiles and methanogens, *etc*., which is consistent with previous reports (Supplementary Table S2)^22^. Distribution patterns of pathways of all five ESMs were analysed in the 427 archaeal species. Phylogenetic analysis was also incorporated into the study in order to have a grouped overview of energy storage metabolisms in archaea.

### Bioinformatics Analysis of PolyP Distribution in Archaea

PolyP has been correlated with prokaryotic adaptation to extreme environments, such as heavy metal and high salinity resistances, *etc.*^6,23–25^. A number of archaeal species, such as *Sulfolobus metallicus* and *Metallosphaera sedula*, were reported to store polyP intracellularly^26^. In this study, a set of eight enzymes that are directly involved in polyP metabolism in prokaryotes was scrutinized in archaeal proteomes. Polyphosphate glucokinase (PPGK) and actin-related protein complex (Arp_C) genes were excluded because only a couple of species have them (data not shown).

As for the six polyP-metabolism related enzymes, PPK1 is responsible for polyP synthesis while the other five enzymes are in the degradation pathway (Fig. 1). Previous study found that PPK1 is widely conserved in bacteria and archaea^27^. In this archaeal study, we observed the co-occurrence of PPK1 and PPK2 (also PAP, a homolog of PPK2 with duplicated domains PF03976) (Supplementary Table S1)^28^, which is consistent with previous bacterial analysis^8,9^. However, PPK1 is only moderately present (99 out of 427 archaeal species) and the combined presence of PPK1 and PPK2 are even more rare in archaea (18 out of 427 archaeal species compared with 504 out of 944 bacterial species)^8^. Technically, coupling PPK1 and any one of the five enzymes (PPK2, PAP, SurE, PPX, and NADK) in archaea could be able to synthesize and degrade polyP (Fig. 1). Thus, a total of eight polyP metabolism groups were analysed, which include Full_Path group (7 archaea) encompassing polyP-related six enzymes (PPK1, PPK2, PAP, SurE, PPX, and NADK), No_Path group (64 archaea) involving species with none of the six enzymes. Functional_Path group (99 archaea) means archaea having PPK1 and at least one polyP-degrading enzyme (PPK2, PAP, SurE, PPX, or NADK). PPK1_PPK2 (18 archaea), PPK1_PAP (30 archaea), PPK1_SurE (88 archaea), PPK1_PPX (39 archaea), and PPK2_NADK (78 archaea) means archaea having PPK1 and the designated polyP-degrading enzyme. Proteome size and GC content were also incorporated into polyP pathway distribution analyses (Fig. 1).

According to the results, presence of Full_Path group may suggest an ancient origin of polyphosphate. In the Full_Path group, the three archaeal genera, *Methanolobus*, *Methanoregula*, and *Methanosarcina*, have complete polyP pathways. It was reported that cells grown on methanol accumulated more phosphate than autotrophically grown cells^29^. Polyphosphate has been frequently observed in *Methanosarcina*, and high concentrations of orthophosphates are associated with acetotrophic methanogenesis^29–31^. In addition, air-adapted *Methanosarcina acetivorans* has both enhanced methane production and polyP accumulation^32^. However, there are no definitive physiological roles for polyP in archaea for methane production^29^. On the other hand, much experimental evidence links polyphosphate with adaptation to extreme environments, such as hyperthermal, heavy metal concentration and hyper-salinity, *etc*., which suggests potential roles of polyP in methanogens as a contributor to multiple extremophilic phenotypes^6,24,33,34^. As for high salinity resistance, phosphate transporter operon (*pst*) was found to be highly induced under high salt concentration in *Methaonsarcina mazei*^35^. Together with polyphosphate, they might serve as counterions for potassium ions, hence increasing resistance to high salinity conditions^35^. In the presence of copper or other heavy metals, polyP can be rapidly degraded into phosphate in species like *Sulfolobus metallicus* by exopolyphosphatase, the first polyP enzyme identified in archaea^26^. The phosphate then forms metal–phosphate complexes and is excreted through inorganic phosphate transport system (Pit)^34,36^. In addition, NAD kinase and PPX may also serve a similar role for heavy metal resistance in *Metallosphaera sedula*^36^.

64 out of 427 archaea (No_Path group) were found having none of the six enzymes. For the previous bacteria study, polyP metabolism loss was linked with reduced proteome size and could be an indicator for obligate symbiotic, parasitic or commensal (SPC) lifestyle^8,37^. Reduced proteome was also observed in archaeal No_Path group (Fig. 1). However, archaea are almost exclusively free-living species and SPC archaea are rarely reported^38^. Thus, their proteome reduction could not be interpreted by genetic drift with increased non-coding genes and loss of biosynthetic pathways^37^. Loss of polyP enzymes or the whole pathway could be caused by minimization of cell complexity under extreme environments, where polyP metabolism might not be necessary^37^. As for the functional pathway groups, it is interesting to notice that all species with PPK1 are coupled with at least one polyP degrading enzyme. In addition, proteome sizes and GC contents of the PPK1_PPK2, PPK2_SurE and PPK1_NADK groups are similar (Fig. 1). In contrast, PPK1_PAP and PPK1_PPX are distinct due to their comparatively smaller genome size and lower GC content (Fig. 1). In addition, PAP (polyphosphate: AMP Phosphotransferase) and PPX (exopolyphosphatase) seem to be more closely associated with methanogenic archaea than other enzymes.

Intriguingly, some archaeal species that are methanogenic, halophilic or thermophilic such as *Metallosphaera sedula* and *Sulfolobus sp*. have been reported to accumulate polyP experimentally^26^. However, enzyme screening via HMM-based sequence models did not identify polyP synthesis enzyme PPK1 (Supplementary Table S1). In addition, it was also reported that polyphosphate synthesis enzymes have not yet been described for *Crenarchaeota*, neither, though polyP granules were found in the cytoplasm and could be rapidly hydrolysed to inorganic phosphate^39^. It has been suggested that there might be an unknown archaeal PPK enzyme^40^. This is probably the reason that HMM-based sequence search cannot find homologs. Thus, further experimental studies should be performed to identify the novel enzyme(s). In addition, polyP synthesis pathways seem to be mainly restricted to halophiles and methanogens. Considering that horizontal gene transfer (HGT) from bacteria to halophiles and methanogens is well known^41^, it could be possible that these archaea acquired the respective genes related to polyP metabolism from bacteria whereas other archaea might use so far unknown pathways.

### Bioinformatics Analysis of Glycogen Distribution in Archaea

Presence of glycogen has been reported decades ago in some archaeal genera as *Thermococcus*, *Sulfolobus*, *Thermoproteus*, *and Desulfurococcus*^42^. In addition, *Methanogens* such as *Methanosarcina thermophila* were also found to accumulate glycogen^43^. Although some species belonging to the above-mentioned archaeal genera were reported to accumulate glycogen, it doesn’t mean that all species in these archaeal groups should have the same feature. In addition, we only studied 427 reference archaeal proteomes, it is not supposed to cover all species in archaea. So far, three representative glycogen metabolism pathways have been proposed, which are classical pathway (CP)^44^, non-classical (NCP I) trehalose-related pathway^45^ and non-classical (NCP II) liposaccharide-related pathway (Fig. 2)^46^. For CP, there are three archaea-specific enzymes corresponding to their bacterial counterparts, which are archaeal GlgB, GlgP and GlgX (or TreX)^47^. Archaeal GlgB and GlgP are vastly different from bacterial GlgB and GlgP according to HMM-based Pfam analysis (Table 1)^47,48^. Although GlgX and TreX are homologous with high similarity, their actual functions are rather divergent^49^. HMM-based sequence search showed that they share an identical distribution pattern in selected archaeal proteomes (Supplementary Table S1). Thus, at sequence level we cannot distinguish TreX and GlgX. As for the NCP I, only one archaeal species *Picrophilus torridus* DSM 9790 was previously identified to harbour all four enzyme TreS, Pep2, GlgE and GlgB, which was also confirmed not to be caused by horizontal gene transfer^11^. Finally, Rv3032, the essential enzyme in the other non-classical alpha-glucan metabolism pathway, is responsible for the synthesis of linear alpha-1,4-glycosidc chains^50^.

**Table 1:** Enzymes directly associated with the metabolism of five energy storage compounds (polyphosphate, glycogen, polyhydroxyalkanoate, triacylglycerol and wax ester) in microorganisms.

	Reference Species	D^#^	Gene	Protein	Enzyme	L^#^	UPID^#^	PID^#^
Polyphosphate (polyP)	*Haladaptatus paucihalophilus*	A	*ppk1*	PPK1	Polyphosphate kinase	707	E7QTB5	PF13089 PF02503 PF13090
	*Thiomicrospira cyclica*	A	*ppk2*	PPK2	Polyphosphate kinase 2	264	F6DAB2	PF03976
	*Methanohalophilus mahii*	A	*pap*	PAP	Polyphosphate: AMP phosphotransferase	503	D5EAS0	PF03976 PF03976
	*Haloarcula marismortui*	A	*surE*	SurE	5′-nucleotidase	269	Q5V4L7	PF01975
	*Metallosphaera sedula*	A	*gppA*	GPPA	Ppx/GppA phosphatase	420	A4YFE8	PF02541
	*Pyrococcus horikoshii*	A	*ppnK*	PPNK	NAD kinase	277	O58801	PF01513
	*Mycobacterium tuberculosis*	B	*ppgK*	PPGK	Polyphosphate glucokinase	265	P9WIN1	PF00480
	*Dictyostelium discoideum*	E	*arp*	Arp_C	Actin-related protein (Complex)	/	/	PF00022
Glycogen	*Haloferax massiliensis*	A	*glgC*	GlgC	Glucose-1-phosphate adenylyltransferase	322	A0A0D6JRD4	PF00483
	*Pyrococcus abyssi*	A	*glgA*	GlgA	Glycogen synthase	437	Q9V2J8	PF08323 PF00534
	*Thermococcus kodakaraensis*	A	*glgB*	GlgB	1,4-alpha-glucan branching enzyme (GH57)	675	Q5JDJ7	PF03065 PF09210 PF14520
	*Escherichia coli*	B	*glgB*	GlgB	1,4-alpha-glucan branching enzyme (GH13)	728	P07762	PF02922 PF00128 PF02806
	*Thermococcus gammatolerans*	A	*glgP*	GlgP	Alpha-glucan phosphorylase (DUF3417)	826	C5A1K5	PF11897 PF00343
	*Escherichia coli*	B	*glgP*	GlgP	Alpha-glucan phosphorylase	815	P0AC86	PF00343
	*Sulfolobus solfataricus*	A	*treX*	TreX	Glycogen debranching enzyme	718	Q7LX99	PF02922 PF00128 PF02806
	*Escherichia coli*	B	*glgX*	GlgX	Glycogen debranching enzyme	657	P15067	PF02922 PF00128 PF02806
	*Mycobacterium tuberculosis*	B	*treS*	TreS	Trehalose synthase/amylase	601	P9WQ19	PF00128 PF16657
	*Mycobacterium tuberculosis*	B	*pep2*	Pep2	Maltokinase	455	Q7DAF6	/
	*Picrophilus torridus*	A	*glgE*	GlgE	Alpha-1,4-glucan: maltose-1-phosphate maltosyltransferase	630	Q6L2Z8	PF11896 PF00128
	*Mycobacterium tuberculosis*	B	*Rv3032*	Rv3032	Glycogen synthase	414	P9WMY9	PF13439 PF00534
Polyhydroxyalkanoates (PHAs)	*Haloferax mediterranei*	A	*phaA*	PhaA	Beta-ketothiolase	383	I3R3D1	PF00108 PF02803
	*Haloarcula hispanica*	A	*phaB*	PhaB	Acetoacetyl-CoA reductase	247	E1U2R6	PF00106 PF08659 PF13561
	*Haloarcula hispanica*	A	*phaC*	PhaC	PHA synthase subunit C	474	G0HQZ6	PF07167 PF00561 PF06850 PF14520
	*Haloarcula hispanica*	A	*phaE*	PhaE	PHA synthase subunit E	181	G0HQZ5	PF09712
	*Haloferax mediterranei*	A	*phaP*	PhaP	PHA granule-associated protein	154	I3R9Z2	/
	*Burkholderia pseudomallei*	B	*phaZ*	PhaZ	Intracellular polyhydroxyalkanoate depolymerase	423	Q3JIM5	PF06850
	*Rhizobium fredii*	B	*phaZ*	PhaZ	Polyhydroxyalkanoate depolymerase	369	G9AII6	PF10503
Triacylglycerol (TAG)	*Saccharomyces cerevisiae*	E	*PDAT*	PDAT	Phospholipid: diacylglycerol acyltransferase	661	P40345	PF02450
	*Acinetobacter baylyi*	B	*wax-dgaT*	WS/DGAT	Wax Ester Synthase/Acyl Coenzyme A: Diacylglycerol Acyltransferase	458	Q8GGG1	PF03007 PF06974
	*Pseudomonas aeruginosa*	B	*lip*	Lip	Triacylglycerol lipase	311	P26876	PF00561
Wax Ester (WE)	*Acinetobacter baylyi*	B	*wax-dgaT*	WS/DGAT	Wax Ester Synthase/Acyl Coenzyme A: Diacylglycerol Acyltransferase	458	Q8GGG1	PF03007PF06974

^#^D: three life domains: Archaea (A), Bacteria (B) and Eukaryote (E). L: Protein length. UPID: UniProt Identifier. PID: Pfam Identifier.

Glycogen metabolism distribution in archaea is vastly different from that in bacteria. Among 1202 studied bacterial proteomes, we observed that 402 strains (245 species) have a complete set of essential enzymes (GlgC, GlgA, GlgB, GlgP, and GlgX) for glycogen metabolism^1^. Our analysis showed that no archaeon harbours the complete classic pathway (CP) of glycogen metabolism with all five essential enzymes. Thus, we focused only on glycogen synthesis pathways. In this study, we divided glycogen metabolism into eight groups, which are CP Full Synthesis (Archaea), CP Full Synthesis (Bacteria), CP Null Synthesis, CP GlgP, CP TreX/GlgX, NCP I Synthesis, NCP II Synthesis (Archaea), NCP II Synthesis (Bacteria) (Fig. 2). CP Full Synthesis is a pathway with GlgC, GlgA and GlgB in an archaeal proteome. However, because there are two types of GlgB, we used archaea and bacteria to distinguish the two pathways. From our study, archaeal GlgB (Q5JDJ7) and bacterial GlgB (P07762) do not occur in the same archaeal proteome, and CP Full Synthesis (Archaea) pathway (average proteome size 2214, GC content 47.24%) is closely linked with thermophiles. As for the 17 archaea in the CP Full Synthesis (Archaea) group, most of which belongs to *Thermococci* class. As for physiological traits, they all are thermophilic archaea except for Candidatus *Lokiarchaeota* archaeon CR_4 whose lifestyle is not clear. On the other hand, only five species possess CP Full Synthesis (Bacteria) pathway (average proteome size 2220, GC content 48.36%), three of which are methanogenic. In fact, both methanogenic and thermophilic archaea have been reported to accumulate glycogen^51^. Recently, central carbon mechanism in *Methanosarcina acetivorans* was systematically investigated in order to optimize methane production^52^. Glycogen was experimentally identified and its biological function was considered to be an environmental advantage for *Methanosarcinales* when carbon sources are scarce^52^. However, no halophilic archaea were reported to store glycogen by far. On the other hand, most of the archaea in the CP Null Synthesis group possess the sugar-fermenting trait, though a couple of thermophilic archaea are also present. All the sugar-fermenting archaea in this study belong to the candidate division MSBL1 from the unclassified Euryarchaeota. It was suggested that MSBL1 archaea could ferment glucose via the Embden–Meyerhof–Parnas pathway and were also capable of autotrophic growth when glucose and other fermentable sugars are not available^53^. Thus, there might be no need for these archaea to store glycogen as energy reserve.

There are another two non-classical pathways (NCP) for glycogen synthesis. Although only one archaeon was previously reported to have the complete NCP I pathway, in this study, more species were identified to use the pathway to synthesize alpha-glucan, such as *Acidiplasma cupricumulans* BH2 and *Thermplasmatales* archaeon, etc. Considering that large number of archaeal genomes are yet to be discovered, it could be possible that Tres-Pep2-GlgE-GlgB pathway might be widespread in archaeal domain, contradictory to current view that the pathway is mainly present in bacteria^11^. As for the NCP II synthesis pathway, it has three enzymes GlgC, Rv3032 and GlgB (GH57) and is mainly associated with thermophilic lifestyle (Fig. 2). As for all glycogen-related enzymes, Rv3032 is most widely distributed and 290 archaea has homologs of this enzyme in their proteomes, which suggested that this enzyme might be centric to glycogen formation in archaea.

Degrading enzymes exist in classical glycogen metabolism pathway, which are glycogen phosphorylase (GlgP) and glycogen debranching enzyme (TreX or GlgX). 69 archaea have GlgP enzyme while only 16 archaea have TreX or GlgX enzyme. Since TreX and GlgX are highly similar, HMM-based sequence search cannot distinguish the two proteins. According to Fig. 2, it becomes obvious that many archaea harbour glycogen degrading enzymes but much fewer the biosynthesis pathways. On our data, GlgP is more widespread in archaea than GlgX or TreX. For detailed illustration, please refer to Fig. 2. As for the CP Null Synthesis pathway, there are 21 archaea in this group. These have a small average proteome size of 800 proteins per proteome. Thus, consistent with other two storage compounds, loss of energy reserve ability may lead to a reduced genome size, although the specific mechanisms could be different^37^.

### Bioinformatics Analysis of PHA Distribution in Archaea

Polyhydroxyalkanoic acids (PHAs) are a complex class of biodegradable polyesters found in a wide range of microorganisms, among which short-chain-length (SCL) polyhydroxybutyrate (PHB), poly-3-hydroxyvalerate (PHV) and poly-3-hydroxybutyrate-co-3-hydroxyvalerate (PHBV) are the three main components synthesized in archaea^16^. Currently, researchers are mainly interested in the industrial production and biotechnological applications of the biodegradable PHAs from archaea^54^. Biological functions of PHAs in archaea are less focused. It has been known that PHAs are normally stored in archaea as intracellular energy storage compound and have many potentials in medical applications^55^. Key enzymes involved in PHA biosynthesis include PhaA, PhaB, PhaC and PhaE (Fig. 3)^16^. In addition, PhaP, a PHA-related phasin, was also found to be an essential protein in PHA formation, which is responsible for PHA accumulation and granule morphology^17^.

Pathway analysis in 427 archaea showed that 51 species have complete synthesis pathway for PHA accumulation while 31 species have none of the enzymes (Supplementary Table S1). All of the 51 species with complete PHA synthesis pathway were halophiles, which indicates that PHA accumulation is associated with halophilic phenotype. A recent review also stated that PHA production in archaea has been limited to *Haloarchaeal* species^13^. Although PHA was widely studied in halophilic archaea for industrial purposes, its contributions to archaeal physiology were rarely investigated. Several bacterial studies provided evidences to support the protective roles that PHA might play for archaea under stress conditions, such as UV irradiation, heat, osmotic shock, desiccation and oxidative stress. For example, it has been reported that disruption of the polyhydroxyalkanoate synthase (PhaC) gene in *Aeromonas hydrophila* reduces its survival ability under stress conditions including high osmotic pressure^56^. The study suggested that RNA polymerase sigma S (RpoS) played an important regulatory role in the enhanced resistance of the species to stresses conferred by PHBHHx, a copolymer consisting of 3-hydroxybutyrate and 3-hydroxyhexanoate^56^. However, molecular mechanisms for preferred accumulation of PHAs in halophilic archaea and how PHA contributes to archaeal adaptation in high salinity environments are still lacking and require further experimental exploration. As for the 31 species with no PHA synthesis enzymes, they are associated with different types of archaeal physiological phenotypes and no specific correlational pattern is identified (Fig. 3). Consistently, their proteome sizes are significantly reduced on average (*P*-value < 0.05).

### TAG and WE Metabolisms in Archaea

Currently, TAG is mainly distributed in members of the prokaryotic *Actinomycetes*, while WE is more closely associated with marine or aquatic organisms such as *Marinobacter* or *Acinetobacter*, *etc.*^57–59^. TAG and WE distributions in archaea are less investigated systematically due to experimental limitations and genome availability. Initial scanning for 18 archaeal genome sequences failed to identify any homologues of bifunctional wax ester synthase/acyl-CoA: Diacylglycerol acyltransferase^18^. Until now, TAG accumulation in archaea has not been reported^60^. In this study, all essential enzymes for direct metabolism of both wax ester (*wax-dgaT*) and triacylglycerol (*PDAT*, *lip* and *wax-dgaT*) are not identified in 427 archaeal proteomes through different bioinformatic methods, such as hmmscan, phmmer, and jackhmmer, which confirmed previous conclusion that archaea do not accumulate TAG and WE. However, our study cannot exclude the possibility that other unrecognized pathways might exist, which might be responsible for synthesizing or utilizing the two neutral lipids.

### Phylogenetic Analysis of ESM in Archaea

**Figure 6:**
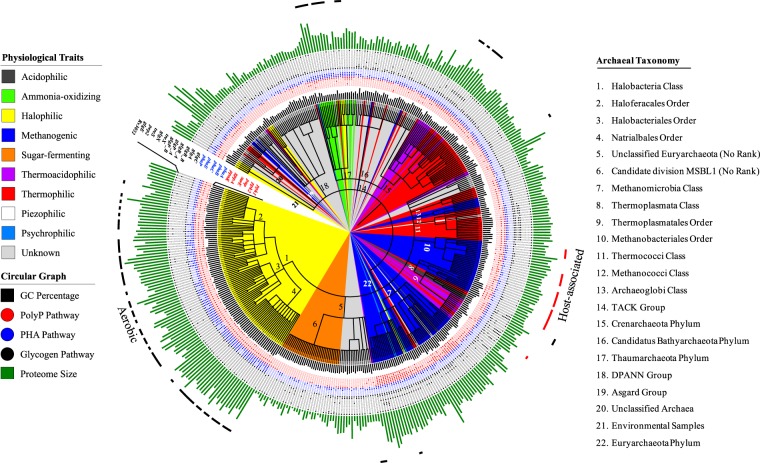
Overview of distribution patterns of all enzymes directly linked with PolyP (red dot), PHA (blue dot), and glycogen (black dot) metabolism. The taxonomy identifier based phylogenetic tree is coloured based on archaeal physiology such as acidophilic (dark grey), halophilic (yellow) and thermophilic (red), *etc*. For the circular graph from inside out, they are GC percentage (black bars), PolyP metabolism (*ppk1*, *ppk2*, *pap*, *surE*, *gppA*, *ppnK*), PHA metabolism enzymes (*phaA*, *phaB*, *phaC*, *phaE*, *phaP*), glycogen metabolism enzymes (*glgC*, *glgA*, *glgB_B*, *glgB_A*, *glgP_A*, *glgP_B*, *treX*, *glgX*, *treS*, *pep2*, *glgE*, *Rv3032*) and proteome sizes (green bars). Blank and filled dots mean loss and gain of corresponding enzymes. For each dot circle, corresponding gene name was given. The outmost two dashed circles represent two phenotypes, aerobic lifestyle (black) and host-associated lifestyle (red).

Archaeal phylogenetic tree constructed from NCBI taxonomy identifiers provided a clustered overview of species with a variety of physiological from halophiles, to methanogens and to thermophiles (Fig. 6). Halophiles are correlated with higher GC content and larger proteome size, while thermophilic archaea normally have small genome sizes and lower GC content. Thus, it is consistent with previous reports that high temperature leads to small genome size, but high GC content is not correlated with thermal stability^61^. In contrast, genomes with high GC content may be more stable in high salinity environments. From the distribution patterns of the five energy storage metabolism pathways, we can find that polyP metabolism is important in methanogens while PHA metabolism plays important roles in halophiles. As for glycogen metabolism, it is possible that it is correlated with thermophilic and thermoacidophilic archaea. Another feature presented in Fig. 6 that is worth of mentioning is that halophiles are mainly associated with aerobic lifestyles (outermost black circle)^62^, while host-associated archaea are mainly found among the methanogens (outermost red circle)^63^.

At this point, archaea are still ecological dark matter due to being unculturable and living in inhospitable environments. Evolutionary study of archaea is still at the infant stage due to the limited number of completely sequenced and assembled archaeal genomes. Insights from our analyses of the 427 archaeal proteomes in this study are helpful for a better understanding of the common features in archaeal genomes and their physiology. With more archaeal genomes available in near future, there will be a much clearer picture about archaeal metabolism, physiology, and adaptation to extreme niches.

## Methods and Materials

### Collection of Archaean and Bacterial Proteomes

A total of 428 archaeal reference proteomes were downloaded from UniProt database via website scraping^20^. Proteome UP000053961 (corresponding to taxon 301375, *Methanosaeta harundinacea*) was removed from the analysis list due to its inconsistency during construction of phylogenetic tree trough phyloT (https://phylot.biobyte.de/). Thus, 427 archaea were included in this study. All proteome file names were renamed with their unique UniProt proteome identifiers. Associated information such as corresponding names, NCBI taxonomy identifiers^64^, proteome sizes (proteins per proteome) and enzyme distributions related to five energy storage compounds were all included in Supplementary Table S1 for further analysis.

### Collection of Metabolism Pathways

Pathways of five energy storage compounds were selected for thorough screening in archaeal and bacterial proteomes: polyP (involving genes *ppk1*, *ppk2*, *pap*, *surE*, *gppA*, *ppnK*, *ppgK*, and *arp*), glycogen (*glgC*, *glgA*, *glgB*, *glgP*, *treX*, *glgX*, *treS*, *pep2*, *glgE* and *Rv3032*), PHAs (*phaA*, *phaB*, *phaC*, *phaE*, *phaP* and *phaZ*), WE (*wax-dgaT*) and TAG (*PDAT*, *wax-dgaT* and *lip*). All enzymes directly linked with the metabolism of the five energy storage compounds were collected and are summarized in Table 1, together with gene names, protein names, original source, enzyme length, UniProt ID and Pfam ID.

### *Ab initio* Construction of Hidden Markov Model (HMM)

Seed sequences of proteins directly linked to the metabolism of energy storage compounds were selected by mining literature and searching UniProt database, focusing on archaeal species^20^. For those enzymes with no homologous sequences in archaea, bacterial counterparts were used. When archaea and bacteria have non-homologous enzymes for the same function, both enzymes were included as seed proteins for HMM constructions, such as GH57 and GH13 GlgB. A complete list of the collected proteins is presented in Table 1. After obtaining sequences for all seed proteins, position specific iterated BLAST (PSI-BLAST) was performed to collect homologous sequences for each seed protein from the NCBI non-redundant (nr) database of protein sequences (Max targe sequence number = 1000, E-value < 0.001) except for GlgB, GlgP, GlgX, PhaP and PhaE^65,66^. 5000 homologues of the five enzymes were collected due to their high conservation. Perl script nrdb90.pl was used to remove 90% or higher similar sequences^67^. The standalone command-line version of MUSCLE was used so the MSAs were done automatically^68^. Heads or tails of multiple sequence alignments tend to be more inconsistent^69^. Thus, all MSAs were manually edited to remove heads and tails by using JalView^70^. The HMMER package was used for *ab initio* construction of HMMs through the hmmbuild command by using multiple sequence alignments^71^. All MSAs results were converted from FASTA to STOCKHOLM format before construction by using Biopython command *SeqIO*.*convert*^72^. A total of 427 archaeal reference proteomes were scanned for the presence of the collected seed proteins. All distribution patterns of enzymes across species were recorded in Supplementary Table S1.

### Proteome Screening for Enzyme Distributions

In order to make sure that all homologous sequences of corresponding enzyme for energy storage compounds were identified, several different methods were initially used to compare the results of archaeal and bacterial proteome searching: full-length hidden Markov models, phmmer (similar to BLASTP), jackhmmer (similar to PSI-BLAST), and concatenated Pfam domains. Only full-length HMM results were analysed in this study. The other three methods turned out to be overly restrictive or overly loose when compared with HMM methods. For the screening results, E-value was set to 0.001 and minimal length of hit sequences was 60% of query sequences in order to get rid of fragmentary sequences. Pfam server (http://pfam.xfam.org/) was used to double-check domain organizations of hit sequences whenever suitable.

### Evolutionary Analysis

NCBI taxonomy identifiers were obtained from UniProt database, which were later used for constructing a phylogenetic tree via phyloT (https://phylot.biobyte.de/about.cgi) in order to investigate distribution patterns of energy storage metabolism in evolutionary settings. phyloT is an online server, using NCBI taxonomy identifier to generate a pruned tree automatically in user-defined output format. Tree visualization and annotation were undertaken through the freely available online editing tool iTOL, with pre-defined tol_binary and tol_simple_bar templates, *etc.*^73^. Distribution patterns of ESMs are added into the phylogenetic tree as circular dot plots in order to identify how energy reserves are distributed in evolution.

### Statistical Analysis

Python scripts and R programming, especially the ggplot2 package, were used throughout the study for data visualization (R Core Team, 2015). Unpaired two-tailed Student’s t-test was used for statistical analysis. Significant difference was defined as p-value less than 0.05.

## Supplementary information

Supplementary Table S2

Supplementary Table S1

## Data Availability

All data generated or analysed during this study are included in this published article (and its Supplementary Information files).
