# Supplementary material for: Bioinformatics Analysis of Metabolism Pathways of Archaeal Energy Reserves
Source: Sci Rep. 2019 Jan 31;9:1034. doi: 10.1038/s41598-018-37768-0 (PMC6355812; doi:10.1038/s41598-018-37768-0)
Supplement: Supplementary file 1 — Supplementary Table S2 [file 41598_2018_37768_MOESM1_ESM.docx]

**Title**

Bioinformatics Analysis of Metabolism Pathways of Archaeal Energy Reserves

**Authors**

Liang Wang^1,2*^, Qinghua Liu^2^, Xiang Wu^1^, Yue Huang^1^, Michael J. Wise^3,4^, Zhanzhong Liu^5^, Wei Wang^1,6,7^, Junfeng Hu^1,8^, Chunying Wang^5^

**Supplementary Table 2** Summary of 427 archaeal species with manually checked information from literature and public databases. -: Not Available, A: Aerobic, AC: Acidophilic, AN: Anaerobic, AO: Ammonia-oxidizing, AQ: Aquatic, FA: Facultative Aerobic, GC%: GC Content Percentage, H: Halophilic, Ha: Habitat, Ho: Host, I: Isolation Country, IS: Isolation Site, LS: Lifestyle, M: Methanogenic, O: Oxygen Requirements, P: Physiology, PP: Psychrophilic, PS: Proteome Size, PZ: Piezophilic, Ref: References, S: Specialized, SF: Sugar-fermenting, T: Thermophilic, TA: Thermoacidiphilic, Taxon: Taxonomy Identifier, Tp: Temperature.

| **Species Names** | **Taxon** | **PS** | **GC%** | **I** | **Tp** | **O** | **Ha** | **IS** | **P** | **LS** | **Ref** |
| --- | --- | --- | --- | --- | --- | --- | --- | --- | --- | --- | --- |
| *Acidianus hospitalis* W1 | 933801 | 2329 | 34.10 | - | 65-95 | FA | AQ | Fresh water | TA | Chemolithoautotrophic | (1) |
| *Acidianus manzaensis* strain YN-25 | 282676 | 2641 | 30.62 | China | 80 | A | AQ | Acid hot spring | TA | - | (2) |
| *Acidilobus saccharovorans* 345-15 | 666510 | 1499 | 57.20 | Russia | 80-85 | AN | S | Solfataric field | TA | Organotrophic | (3) |
| *Acidiplasma cupricumulans* strain BH2 | 312540 | 1683 | 34.17 | Myanmar | 22-63 | A | S | Chalcocite bioleach heap | AC | Chemomixotrophic | (4) |
| *Aciduliprofundum boonei* T469 | 439481 | 1539 | 39.10 | Pacific Ocean | 60-75 | AN | AQ | Acidic hydrothermal vent | TA | Heterotrophic | (5) |
| *Aeropyrum pernix* K1 | 272557 | 1700 | 56.30 | Japan | 95 | A | AQ | Coastal solfatara | T | - | (6) |
| Arc I group archaeon ADurb1113_Bin01801 | 1706434 | 1596 | 33.33 | United States | - | AN | AQ | Digester sludge | M | - | - |
| Arc I group archaeon BMIXfssc0709_Meth_Bin006 | 1706438 | 1729 | 34.49 | United States | - | - | S | Wastewater treatment bioreactor | M | - | - |
| Arc I group archaeon U1lsi0528_Bin055 | 1705409 | 2291 | 35.38 | United States | - | - | S | Wastewater treatment bioreactor | M | - | - |
| Arc I group archaeon U1lsi0528_Bin089 | 1705564 | 1961 | 34.07 | United States | - | - | S | Wastewater treatment bioreactor | M | - | - |
| *Archaeoglobus fulgidus* DSM 4304 | 224325 | 2399 | 48.60 | Italy | 60-95 | AN | AQ | Geothermally heated sea floor | T | Organoheterotrophic/Lithoautotrophical | (7) |
| *Archaeoglobus profundus* DSM 5631 | 572546 | 1822 | 42.00 | Mexico | 90 | AN | AQ | Marine hydrothermal system | T | Mixotrophic | (8) |
| *Archaeoglobus sulfaticallidus* PM70-1 | 387631 | 2213 | 43.20 | Pacific Ocean | - | - | AQ | Marine hydrothermal system | T | Chemolithoautotrophic | (9) |
| *Archaeoglobus veneficus* SNP6 | 693661 | 2065 | 47.00 | Atlantic Ocean | 65-85 | AN | AQ | Marine hydrothermal system | T | Chemolithoautotrophic | (10) |
| archaeon D22 strain D22 | 1932700 | 474 | 35.52 | South Korea | - | - | S | Tidal flat sediment | - | - | - |
| archaeon GW2011_AR10 | 1579370 | 1339 | 43.06 | United States | - | - | AQ | Groundwater | - | - | - |
| archaeon GW2011_AR15 | 1579373 | 1308 | 42.48 | United States | - | - | AQ | Groundwater | - | - | - |
| archaeon Heimdall AB_125 | 1841596 | 2348 | 33.36 | Denmark | - | - | AQ | Marine sediment | T | - | (11) |
| archaeon Heimdall LC_2 | 1841597 | 4485 | 32.64 | - | - | - | AQ | Hydrothermal vent sediment | T | - | (11) |
| archaeon Heimdall LC_3 | 1841598 | 5374 | 29.72 | - | - | - | AQ | Hydrothermal vent sediment | T | - | (11) |
| archaeon Odin LCB_4 | 1841599 | 1584 | 38.05 | United States | - | - | AQ | Hot spring | T | - | (11) |
| archaeon RBG_16_50_20 | 1797286 | 1297 | 49.96 | United States | - | - | S | Rifle background sediment | - | - | - |
| *Bathyarchaeota archaeon* BA1 | 1700835 | 2403 | 47.07 | Australia | - | AN | S | Coalbed methane well | M | Methylotrophic | (12) |
| *Bathyarchaeota archaeon* BA2 | 1700836 | 1761 | 44.20 | Australia | - | AN | S | Coalbed methane well | M | Methylotrophic | (12) |
| *Caldisphaera lagunensis* DSM 15908 | 1056495 | 1477 | 30.00 | Philippines | 70-85 | AN | AQ | Acidic hot spring | TA | Chemoorganotrophic | (13) |
| *Caldivirga maquilingensis* IC-167 | 397948 | 1962 | 43.10 | Philippines | 60-92 | AN | AQ | Acidic hot spring | TA | Heterotrophic | (14) |
| candidate divison MSBL1 archaeon SCGC-AAA259A05 | 1698259 | 1212 | 46.82 | Saudi Arabia | - | AN | AQ | Deep sea brine | SF | Autotrophic | (15) |
| candidate divison MSBL1 archaeon SCGC-AAA259B11 | 1698260 | 852 | 44.40 | Saudi Arabia | - | AN | AQ | Deep sea brine | SF | Autotrophic | (15) |
| candidate divison MSBL1 archaeon SCGC-AAA259D14 | 1698261 | 885 | 43.42 | Saudi Arabia | - | AN | AQ | Deep sea brine | SF | Autotrophic | (15) |
| candidate divison MSBL1 archaeon SCGC-AAA259D18 | 1698262 | 389 | 46.89 | Saudi Arabia | - | AN | AQ | Deep sea brine | SF | Autotrophic | (15) |
| candidate divison MSBL1 archaeon SCGC-AAA259E17 | 1698263 | 891 | 47.12 | Saudi Arabia | - | AN | AQ | Deep sea brine | SF | Autotrophic | (15) |
| candidate divison MSBL1 archaeon SCGC-AAA259E19 | 1698264 | 1417 | 47.00 | Saudi Arabia | - | AN | AQ | Deep sea brine | SF | Autotrophic | (15) |
| candidate divison MSBL1 archaeon SCGC-AAA259E22 | 1698265 | 843 | 43.71 | Saudi Arabia | - | AN | AQ | Deep sea brine | SF | Autotrophic | (15) |
| candidate divison MSBL1 archaeon SCGC-AAA259I07 | 1698266 | 671 | 43.36 | Saudi Arabia | - | AN | AQ | Deep sea brine | SF | Autotrophic | (15) |
| candidate divison MSBL1 archaeon SCGC-AAA259I09 | 1698267 | 1400 | 44.42 | Saudi Arabia | - | AN | AQ | Deep sea brine | SF | Autotrophic | (15) |
| candidate divison MSBL1 archaeon SCGC-AAA259I14 | 1698268 | 774 | 43.10 | Saudi Arabia | - | AN | AQ | Deep sea brine | SF | Autotrophic | (15) |
| candidate divison MSBL1 archaeon SCGC-AAA259J03 | 1698269 | 964 | 44.97 | Saudi Arabia | - | AN | AQ | Deep sea brine | SF | Autotrophic | (15) |
| candidate divison MSBL1 archaeon SCGC-AAA259M10 | 1698270 | 893 | 44.22 | Saudi Arabia | - | AN | AQ | Deep sea brine | SF | Autotrophic | (15) |
| candidate divison MSBL1 archaeon SCGC-AAA259O05 | 1698271 | 1138 | 48.14 | Saudi Arabia | - | AN | AQ | Deep sea brine | SF | Autotrophic | (15) |
| candidate divison MSBL1 archaeon SCGC-AAA261C02 | 1698272 | 703 | 45.71 | Saudi Arabia | - | AN | AQ | Deep sea brine | SF | Autotrophic | (15) |
| candidate divison MSBL1 archaeon SCGC-AAA261D19 | 1698273 | 687 | 45.50 | Saudi Arabia | - | AN | AQ | Deep sea brine | SF | Autotrophic | (15) |
| candidate divison MSBL1 archaeon SCGC-AAA261F17 | 1698274 | 559 | 45.95 | Saudi Arabia | - | AN | AQ | Deep sea brine | SF | Autotrophic | (15) |
| candidate divison MSBL1 archaeon SCGC-AAA261F19 | 1698275 | 687 | 45.31 | Saudi Arabia | - | AN | AQ | Deep sea brine | SF | Autotrophic | (15) |
| candidate divison MSBL1 archaeon SCGC-AAA261G05 | 1698276 | 601 | 45.59 | Saudi Arabia | - | AN | AQ | Deep sea brine | SF | Autotrophic | (15) |
| candidate divison MSBL1 archaeon SCGC-AAA261O19 | 1698277 | 779 | 45.49 | Saudi Arabia | - | AN | AQ | Deep sea brine | SF | Autotrophic | (15) |
| candidate divison MSBL1 archaeon SCGC-AAA382A03 | 1698278 | 427 | 39.22 | Saudi Arabia | - | AN | AQ | Deep sea brine | SF | Autotrophic | (15) |
| candidate divison MSBL1 archaeon SCGC-AAA382A13 | 1698279 | 450 | 39.78 | Saudi Arabia | - | AN | AQ | Deep sea brine | SF | Autotrophic | (15) |
| candidate divison MSBL1 archaeon SCGC-AAA382A20 | 1698280 | 947 | 41.83 | Saudi Arabia | - | AN | AQ | Deep sea brine | SF | Autotrophic | (15) |
| candidate divison MSBL1 archaeon SCGC-AAA382C18 | 1698281 | 551 | 41.23 | Saudi Arabia | - | AN | AQ | Deep sea brine | SF | Autotrophic | (15) |
| candidate divison MSBL1 archaeon SCGC-AAA382F02 | 1698282 | 344 | 41.70 | Saudi Arabia | - | AN | AQ | Deep sea brine | SF | Autotrophic | (15) |
| candidate divison MSBL1 archaeon SCGC-AAA382K21 | 1698283 | 218 | 43.47 | Saudi Arabia | - | AN | AQ | Deep sea brine | SF | Autotrophic | (15) |
| candidate divison MSBL1 archaeon SCGC-AAA382M17 | 1698284 | 356 | 43.40 | Saudi Arabia | - | AN | AQ | Deep sea brine | SF | Autotrophic | (15) |
| candidate divison MSBL1 archaeon SCGC-AAA382N08 | 1698285 | 431 | 40.22 | Saudi Arabia | - | AN | AQ | Deep sea brine | SF | Autotrophic | (15) |
| candidate divison MSBL1 archaeon SCGC-AAA385D11 | 1698286 | 299 | 45.56 | Saudi Arabia | - | AN | AQ | Deep sea brine | SF | Autotrophic | (15) |
| candidate divison MSBL1 archaeon SCGC-AAA385M02 | 1698287 | 156 | 35.09 | Saudi Arabia | - | AN | AQ | Deep sea brine | SF | Autotrophic | (15) |
| candidate divison MSBL1 archaeon SCGC-AAA385M11 | 1698288 | 228 | 46.37 | Saudi Arabia | - | AN | AQ | Deep sea brine | SF | Autotrophic | (15) |
| candidate divison MSBL1 archaeon SCGC-AAA833F18 | 1698257 | 278 | 45.03 | Saudi Arabia | - | AN | AQ | Deep sea brine | SF | Autotrophic | (15) |
| candidate divison MSBL1 archaeon SCGC-AAA833K04 | 1698258 | 196 | 45.88 | Saudi Arabia | - | AN | AQ | Deep sea brine | SF | Autotrophic | (15) |
| *Candidatus Acidianus copahuensis* strain ALE1 | 1160895 | 2328 | 35.60 | Argentina | 75 | A/AN | AQ | Hot spring | TA | Chemolithoautotrophic/Heterotrophic | (16) |
| *Candidatus Aenigmarchaeota archaeon* CG1_02_38_14 | 1803500 | 1011 | 37.51 | United States | - | - | AQ | Groundwater | - | - | - |
| *Candidatus Aenigmarchaeota archaeon* ex4484_14 | 2012502 | 880 | 38.96 | United States | - | - | AQ | Deep-sea hydrothermal vent sediments | - | - | - |
| *Candidatus Aenigmarchaeota archaeon* ex4484_224 | 2012503 | 589 | 27.75 | United States | - | - | AQ | Deep-sea hydrothermal vent sediments | - | - | - |
| *Candidatus Aenigmarchaeota archaeon* ex4484_52 | 2012504 | 621 | 27.95 | United States | - | - | AQ | Deep-sea hydrothermal vent sediments | - | - | - |
| *Candidatus Aenigmarchaeota archaeon* ex4484_56 | 2012505 | 657 | 28.98 | United States | - | - | AQ | Deep-sea hydrothermal vent sediments | - | - | - |
| *Candidatus Altiarchaeales archaeon* A3 strain A3 | 1933927 | 1305 | 31.79 | United States | - | - | S | Microbial mat | - | - | - |
| *Candidatus Altiarchaeales archaeon* ex4484_2 | 2012506 | 1481 | 45.61 | United States | - | - | AQ | Deep-sea hydrothermal vent sediments | - | - | - |
| *Candidatus Altiarchaeales archaeon* ex4484_43 | 2012507 | 1178 | 42.85 | United States | - | - | AQ | Deep-sea hydrothermal vent sediments | - | - | - |
| *Candidatus Altiarchaeales archaeon* ex4484_96 | 2012508 | 1009 | 44.32 | United States | - | - | AQ | Deep-sea hydrothermal vent sediments | - | - | - |
| *Candidatus Altiarchaeales archaeon HGW-Altiarchaeales-1* | 2013673 | 2007 | 32.97 | Japan | - | - | AQ | groundwater | - | - | - |
| *Candidatus Altiarchaeales archaeon HGW-Altiarchaeales-2* | 2013674 | 1751 | 32.7 | Japan | - | - | AQ | groundwater | - | - | - |
| *Candidatus Altiarchaeales archaeon HGW-Altiarchaeales-3* | 2013675 | 608 | 34.64 | Japan | - | - | AQ | groundwater | - | - | - |
| *Candidatus Altiarchaeales archaeon IMC4 strain* IMC4_SM1 | 1878999 | 1328 | 47.88 | Germany | - | - | AQ | Sulfidic groundwater | - | - | - |
| *Candidatus Altiarchaeales archaeon* WOR_SM1_79 | 1849365 | 2705 | 39.76 | United States | - | - | AQ | Methane-rich estuary sediments | - | - | - |
| *Candidatus Altiarchaeales archaeon* WOR_SM1_86-2 | 1849364 | 2066 | 40.83 | United States | - | - | AQ | Sulphate-methane transition zone estuary sediments | - | - | - |
| *Candidatus Altiarchaeales archaeon* WOR_SM1_SCG | 1849261 | 2164 | 37.12 | United States | - | - | AQ | Methane-rich estuary sediments | - | - | - |
| *Candidatus Altiarchaeum sp.* CG2_30_32_3053 | 1803514 | 1351 | 32.19 | United States | - | - | AQ | Groundwater | - | - | - |
| *Candidatus Bathyarchaeota archaeon* B23 | 1779367 | 747 | 61.78 | Mexico | - | - | AQ | Marine sediment | - | - | - |
| *Candidatus Bathyarchaeota archaeon* B24 | 1779368 | 1576 | 51.06 | Mexico | - | - | AQ | Marine sediment | - | - | - |
| *Candidatus Bathyarchaeota archaeon* B24-2 | 2041147 | 1404 | 47.3 | Mexico | - | - | AQ | Deep-sea vent sediments | - | - | - |
| *Candidatus Bathyarchaeota archaeon* B25 | 1779369 | 857 | 50.48 | Mexico | - | - | AQ | Marine sediment | - | - | - |
| *Candidatus Bathyarchaeota archaeon* B26-1 | 1779370 | 1185 | 52.66 | Mexico | - | - | AQ | Marine sediment | - | - | - |
| *Candidatus Bathyarchaeota archaeon* B26-2 | 1779371 | 1630 | 51.91 | Mexico | - | - | AQ | Marine sediment | - | - | - |
| *Candidatus Bathyarchaeota archaeon* B63 | 1779372 | 823 | 53.86 | Mexico | - | - | AQ | Marine sediment | - | - | - |
| *Candidatus Bathyarchaeota archaeon* CG_4_8_14_3_um_filter_42_8 | 1974384 | 886 | 40.86 | United States | - | - | AQ | Groundwater | - | - | - |
| *Candidatus Bathyarchaeota archaeon* CG07_land_8_20_14_0_80_47_9 | 1974385 | 1731 | 47.13 | United States | - | - | AQ | Groundwater | - | - | - |
| *Candidatus Bathyarchaeota archaeon* ex4484_135 | 2012509 | 1083 | 58.77 | United States | - | - | AQ | Deep-sea hydrothermal vent sediments | T | - | - |
| *Candidatus Bathyarchaeota archaeon* ex4484_205 | 2012510 | 1448 | 42.11 | United States | - | - | AQ | Deep-sea hydrothermal vent sediments | T | - | - |
| *Candidatus Bathyarchaeota archaeon* ex4484_218 | 2012511 | 737 | 33.1 | United States | - | - | AQ | Deep-sea hydrothermal vent sediments | T | - | - |
| *Candidatus Bathyarchaeota archaeon* ex4484_231 | 2012512 | 969 | 46.72 | United States | - | - | AQ | Deep-sea hydrothermal vent sediments | T | - | - |
| *Candidatus Bathyarchaeota archaeon* ex4484_40 | 2012513 | 1267 | 51.6 | United States | - | - | AQ | Deep-sea hydrothermal vent sediments | T | - | - |
| *Candidatus Bathyarchaeota archaeon* RBG_13_38_9 | 1797377 | 1550 | 36.77 | United States | - | - | S | Rifle background sediment | - | - | - |
| *Candidatus Bathyarchaeota archaeon* RBG_13_46_16b | 1797378 | 1317 | 45.21 | United States | - | - | S | Rifle background sediment | - | - | - |
| *Candidatus Bathyarchaeota archaeon* RBG_13_52_12 | 1797379 | 1779 | 50.48 | United States | - | - | S | Rifle background sediment | - | - | - |
| *Candidatus Bathyarchaeota archaeon* RBG_13_60_20 | 1797380 | 1254 | 60.82 | United States | - | - | S | Rifle background sediment | - | - | - |
| *Candidatus Bathyarchaeota archaeon* RBG_16_48_13 | 1797381 | 867 | 47.99 | United States | - | - | S | Rifle background sediment | - | - | - |
| *Candidatus Bathyarchaeota archaeon* RBG_16_57_9 | 1797382 | 1359 | 58.26 | United States | - | - | S | Rifle background sediment | - | - | - |
| *Candidatus Bathyarchaeota archaeon* strain NP77 | 2026714 | 1880 | 48.39 | Indian Ocean | - | - | AQ | Marine water sample | - | - | - |
| *Candidatus Caldiarchaeum subterraneum* | 311458 | 2154 | 51.60 | Japan | - | - | S | Subsurface mine microbial mat | T | Heterotrophic | (17) |
| *Candidatus Diapherotrites*  *archaeon* CG_4_10_14_0_2_um_filter_31_5 | 1974402 | 655 | 30.61 | United States | - | - | AQ | Groundwater | - | - | - |
| *Candidatus Diapherotrites archaeon* CG08_land_8_20_14_0_20_30_16 | 1974403 | 782 | 29.67 | United States | - | - | AQ | Groundwater | - | - | - |
| *Candidatus Diapherotrites archaeon* CG08_land_8_20_14_0_20_34_12 | 1974404 | 1017 | 34.25 | United States | - | - | AQ | Groundwater | - | - | - |
| *Candidatus Diapherotrites archaeon* CG09_land_8_20_14_0_10_32_12 | 1974405 | 631 | 31.77 | United States | - | - | AQ | Groundwater | - | - | - |
| *Candidatus Diapherotrites archaeon* CG10_big_fil_rev_8_21_14_0_10_31_34 | 1974406 | 1100 | 30.81 | United States | - | - | AQ | Groundwater | - | - | - |
| *Candidatus Diapherotrites archaeon* CG11_big_fil_rev_8_21_14_0_20_37_9 | 1974407 | 1219 | 37.19 | United States | - | - | AQ | Groundwater | - | - | - |
| *Candidatus Diapherotrites archaeon* strain ARS1427 | 2026736 | 607 | 35.34 | Atlantic Ocean | - | - | AQ | Marine water sample | - | - | - |
| *Candidatus Halobonum tyrrellensis* G22 | 1324957 | 3445 | 70.10 | Australia | - | A | AQ | Hypersaline water | H | - | (18) |
| *Candidatus Haloredivivus sp.* G17 | 1072681 | 2152 | 42.00 | - | - | - | AQ | Hypersaline saltern ponds | H | Photoheterotrophic | (19) |
| *Candidatus Heimdallarchaeota archaeon strain* RS678 | 2026747 | 1353 | 29.98 | Saudi Arabia | - | - | AQ | Marine water sample | - | - | - |
| *Candidatus Korarchaeum cryptofilum* OPF8 | 374847 | 1602 | 49.00 | United States | 85 | AN | AQ | Hot spring | T | Heterotrophic | (20) |
| *Candidatus Lokiarchaeota archaeon* CR_4 | 1849166 | 4413 | 44.04 | United States | - | - | AQ | Rifle background sediment | - | - | - |
| *Candidatus Methanomassiliicoccus intestinalis* Issoire-Mx1 | 1295009 | 1826 | 41.30 | France | 37 | AN | Ho | Enrichment culture from feces | M | Methylotrophic | (21) |
| *Candidatus Methanomethylophilus alvus* Mx1201 | 1236689 | 1643 | 55.60 | France | 37 | AN | Ho | Gut | M | Methylotrophic | (22) |
| *Candidatus Methanomethylophilus sp.* 1R26 | 1769296 | 1473 | 60.39 | Denmark | - | AN | Ho | Rumen fluid | M | Methylotrophic | (23) |
| *Candidatus Methanoperedens nitroreducens* strain ANME-2d | 1392998 | 3430 | 43.20 | Australia | 22-35 | AN | AQ | Freshwater lake sediment | M | Methanotrophic | (24) |
| *Candidatus Methanoperedens sp.* DS-2015 | 1719120 | 4527 | 40.18 | Netherlands | - | AN | AQ | - | - | Methanotrophic | (25) |
| *Candidatus Methanoplasma termitum* MpT1 | 1577791 | 1393 | 49.21 | Germany | - | AN | Ho | Gut | M | Hydrogenotrophic | (26) |
| *Candidatus Micrarchaeota archaeon* CG08_land_8_20_14_0_20_59_11 | 1974414 | 866 | 58.66 | United States | - | - | AQ | Groundwater | - | - | - |
| *Candidatus Micrarchaeota archaeon* CG1_02_47_40 | 1805247 | 1150 | 46.99 | United States | - | - | AQ | Groundwater | - | - | - |
| *Candidatus Micrarchaeota archaeon* CG1_02_49_24 | 1805248 | 1159 | 49.16 | United States | - | - | AQ | Groundwater | - | - | - |
| *Candidatus Micrarchaeota archaeon* CG1_02_51_15 | 1805249 | 1244 | 50.59 | United States | - | - | AQ | Groundwater | - | - | - |
| *Candidatus Micrarchaeota archaeon* CG1_02_55_22 | 1805250 | 1162 | 54.74 | United States | - | - | AQ | Groundwater | - | - | - |
| *Candidatus Micrarchaeota archaeon* CG1_02_55_41 | 1805251 | 768 | 54.6 | United States | - | - | AQ | Groundwater | - | - | - |
| *Candidatus Micrarchaeota archaeon* CG1_02_60_51 | 1805252 | 849 | 59.85 | United States | - | - | AQ | Groundwater | - | - | - |
| *Candidatus Micrarchaeota archaeon* CG10_big_fil_rev_8_21_14_0_10_45_29 | 1974417 | 1144 | 45.13 | United States | - | - | AQ | Groundwater | - | - | - |
| *Candidatus Micrarchaeota archaeon* CG10_big_fil_rev_8_21_14_0_10_59_7 | 1974419 | 960 | 57.91 | United States | - | - | AQ | Groundwater | - | - | - |
| *Candidatus Micrarchaeota archaeon Mia14* strain Mia14 | 1920749 | 944 | 39.36 | United Kingdom | - | - | AQ | Acid mine drainage | - | - | - |
| *Candidatus Micrarchaeota archaeon* RBG_16_36_9 | 1801632 | 791 | 35.36 | United States | - | - | S | Rifle background sediment | - | - | - |
| *Candidatus Micrarchaeota archaeon* RBG_16_49_10 | 1801633 | 992 | 47.27 | United States | - | - | S | Rifle background sediment | - | - | - |
| *Candidatus Micrarchaeum sp*. AZ1 | 1531428 | 840 | 45.83 | Mexico | - | - | S | Thermal fumarole | - | - | - |
| *Candidatus Nitrosoarchaeum koreensis* MY1 | 1001994 | 1945 | 32.70 | - | - | - | S | Soil | AO | Oligotrophic | (27) |
| *Candidatus Nitrosoarchaeum limnia* SFB1 | 886738 | 2038 | 31.92 | United States | - | A | AQ | Estuarine sediments | AO | Chemolithoautotrophic | (28) |
| *Candidatus Nitrosopelagicus brevis* V2 | 1410606 | 1444 | 33.16 | Open Ocean | 22 | - | AQ | Open ocean | AO | Chemolithoautotrophic | (29) |
| *Candidatus Nitrosopumilus koreensis* AR1 | 1229908 | 1890 | 34.20 | Arctic Circle | - | A | AQ | Estuarine sediments | AO | Chemoautotrophical | (30) |
| *Candidatus Nitrosopumilus salaria* BD31 | 859350 | 2154 | 33.80 | United States | - | A | AQ | Estuarine sediments | AO | Chemoautotrophical | (31) |
| *Candidatus Nitrosopumilus sp.* AR2 | 1229909 | 1974 | 33.60 | Arctic Circle | - | A | AQ | Marine sediments | AO | Chemolithoautotrophic | (32) |
| *Candidatus Nitrososphaera gargensis* Ga9.2 | 1237085 | 3523 | 48.30 | Russia | 46 | A | AQ | Hot spring | AO | Chemolithoautotrophic | (33) |
| *Candidatus Nitrosotalea devanaterra* | 1078905 | 2102 | 37.07 | - | - | - | - | - | AO | Autotrophic | (34) |
| *Candidatus Pacearchaeota archaeon* CG1_02_35_32 | 1805297 | 1013 | 35.06 | United States | - | - | AQ | Groundwater | - | - | - |
| *Candidatus Pacearchaeota archaeon* CG10_big_fil_rev_8_21_14_0_10_31_24 | 1974438 | 1115 | 31.27 | United States | - | - | AQ | Groundwater | - | - | - |
| *Candidatus Pacearchaeota archaeon* CG10_big_fil_rev_8_21_14_0_10_34_76 | 1974444 | 1076 | 34.43 | United States | - | - | AQ | Groundwater | - | - | - |
| *Candidatus Parvarchaeum acidophilus* ARMAN-5 | 662762 | 1002 | 34.89 | United States | - | - | AQ | Acid mine drainage | AC | - | - |
| *Candidatus Syntrophoarchaeum butanivorans* strain BOX1 | 1839936 | 1592 | 48.68 | Mexico | - | AN | AQ | Hydrothermal sediments | T | Methanotrophic | (35) |
| *Candidatus Syntrophoarchaeum caldarius* strain BOX2 | 1838285 | 1784 | 45.37 | Mexico | - | AN | AQ | Hydrothermal sediments | T | Methanotrophic | (35) |
| *Candidatus Thorarchaeota archaeon* AB_25 | 1837170 | 2914 | 44.58 | Denmark | - | - | AQ | Marine sediment | - | - | - |
| *Candidatus Woesearchaeota archaeon* CG_4_10_14_0_8_um_filter_47_5 | 1974451 | 1016 | 46.82 | United States | - | - | AQ | Groundwater | - | - | - |
| *Cenarchaeum symbiosum* A | 414004 | 2022 | 57.40 | United States | 7-19 | - | AQ | Sponge | PP | Chemolithotrophic | (36) |
| *Crenarchaeota archaeon* 13_1_20CM_2_51_8 | 1805093 | 3280 | 51.68 | United States | - | - | S | Soil | - | - | - |
| *Crenarchaeota archaeon* 13_1_40CM_3_52_10 | 1805095 | 1408 | 51.92 | United States | - | - | S | Soil | - | - | - |
| *Crenarchaeota archaeon* 13_1_40CM_3_52_17 | 1805096 | 1852 | 51.79 | United States | - | - | S | Soil | - | - | - |
| *Crenarchaeota archaeon* 13_1_40CM_3_52_4 | 1805097 | 791 | 52.06 | United States | - | - | S | Soil | - | - | - |
| *Crenarchaeota archaeon* 13_1_40CM_3_53_5 | 1805098 | 2419 | 52.86 | United States | - | - | S | Soil | - | - | - |
| *Desulfurococcales archaeon* ex4484_217_2 | 2012519 | 2399 | 37.78 | United States | 85-106 | A/AN | AQ | Deep-sea hydrothermal vent sediments | T | Chemolithoautotrophic | (37) |
| *Desulfurococcales archaeon* ex4484_42 | 2012520 | 2046 | 38.39 | United States | 85-106 | A/AN | AQ | Deep-sea hydrothermal vent sediments | T | Chemolithoautotrophic | (37) |
| *Desulfurococcales archaeon* ex4484_58 | 2012521 | 1490 | 34.43 | United States | 85-106 | A/AN | AQ | Deep-sea hydrothermal vent sediments | T | Chemolithoautotrophic | (37) |
| *Desulfurococcus kamchatkensis* 1221n | 490899 | 1470 | 45.30 | Russia | 82 | AN | AQ | Terrestrial hot spring | T | Organotrophic | (38) |
| *Euryarchaeota archaeon* AMET1 strain AMET1 | 1927129 | 1510 | 37.90 | Russia | - | - | AQ | Hypersaline soda lakes | - | - | - |
| *Euryarchaeota archaeon* HMET1 | 1903181 | 2152 | 35.38 | Russia | - | - | AQ | Hypersaline salt lake sediment | - | - | - |
| *Euryarchaeota archaeon* strain ARS80 | 2026739 | 822 | 44.85 | Indian Ocean | - | - | AQ | Marine water sample | - | - | - |
| *Euryarchaeota archaeon* TMED215 strain TMED215 | 1986690 | 865 | 46.58 | - | - | - | AQ | Seawater | - | - | - |
| *Euryarchaeota archaeon* TMED279 strain TMED279 | 1986694 | 851 | 42.96 | - | - | - | AQ | Seawater | - | - | - |
| *Ferroglobus placidus* DSM 10642 | 589924 | 2463 | 44.10 | Italy | 85 | AN | AQ | Submarine hypothermal vent | T | Chemolithotrophic/Chemoorganotrophic | (39) |
| *Ferroplasma acidarmanus fer1* | 333146 | 1927 | 36.50 | United States | 40 | AN | AQ | Slime streamers | AC | Oligotrophic | (40) |
| *Ferroplasma sp.* Type II | 261388 | 2671 | 36.45 | United States | - | - | AQ | Acid mine drainage | AC | - | - |
| *Fervidicoccus fontis* Kam940 | 1163730 | 1384 | 37.50 | Russia | 55-85 | AN | AQ | Hot spring | T | Organotrophic | (41) |
| *Geoglobus ahangari* strain 234 | 113653 | 1973 | 53.11 | United States | 88 | AN | AQ | Marine hydrothermal vent | T | Autotrophic | (42) |
| *Hadesarchaea archaeon* CG08_land_8_20_14_0_20_51_8 | 1975561 | 721 | 50.88 | United States | - | - | AQ | Groundwater | - | - | (43) |
| *Hadesarchaea archaeon* DG-33 | 1775754 | 862 | 50.46 | United States | - | - | AQ | Methane-rich estuary sediments | - | - | - |
| *Hadesarchaea archaeon* DG-33-1 | 1775755 | 951 | 51.22 | United States | - | - | AQ | Methane-rich estuary sediments | - | - | - |
| *Hadesarchaea archaeon* YNP_45 | 1776334 | 1281 | 54.68 | United States | - | - | AQ | hot spring sediments | - | - | - |
| *Hadesarchaea archaeon* YNP_N21 | 1776333 | 1227 | 48.65 | United States | - | - | AQ | hot spring sediments | - | - | - |
| *Haladaptatus litoreus* strain CGMCC 1.7737 | 553468 | 4885 | 56.99 | China | 37-40 | A | AQ | Marine solar saltern | H | Chemoorganotrophic | (44) |
| *Haladaptatus sp.* R4 | 1679489 | 4086 | 60.18 | India | - | - | AQ | Saltern | H | - | (45) |
| *Halalkalicoccus jeotgali* B3 | 795797 | 3779 | 62.56 | Korea | 21-50 | - | S | Fermented seafood | H | - | (46) |
| *Halanaeroarchaeum sulfurireducens* strain HSR2 | 1604004 | 2225 | 55.41 | Russia | 15-50 | AN | AQ | Anoxic sediment | H | - | (47) |
| *Halarchaeum acidiphilum* MH1-52-1 | 1261545 | 2706 | 67.40 | Australia | 15-45 | A | S | Solar salt | H | Chemoorganotrophic | (48) |
| *Haloarchaeobius iranensis* strain EB21, IBRC-M 10013, KCTC 4048 | 996166 | 3852 | 67.31 | Iran | 25-50 | A | AQ | Salt lake | H | - | (49) |
| *haloarchaeon* HSR6 | 1873524 | 2099 | 63.62 | Russia | 37-50 | AN | AQ | Hypersaline lake | H | Lithoheterotrophic | (50) |
| *Haloarcula marismortui* ATCC 43049 | 272569 | 4234 | 61.14 | Israel | 40-50 | A | AQ | Dead Sea | H | - | (51) |
| *Halobacteriaceae archaeon* SB9 | 1514971 | 3761 | 62.53 | Saudi Arabia | - | - | AQ | Brine seawater interface | H | - | - |
| *Halobacterium hubeiense* strain JI20-8 | 1407499 | 3340 | 66.24 | China | 22-50 | A | S | Rock salt | H | - | (52) |
| *Halobacterium jilantaiense* strain CGMCC 1.5337 | 355548 | 3054 | 67.93 | China | 22-55 | A | AQ | Salt lake | H | Chemoorganotrophic | (53) |
| *Halobacterium sp.* DL1 | 751944 | 3265 | 66.45 | - | - | - | - | - | H | - | - |
| *Halobacterium sp.* NRC-1 | 64091 | 2426 | 65.92 | United States | 42 | FA | AQ | Salt lake | H | Phototrophic | (54) |
| *Halobaculum gomorrense* strain DSM 9297 | 43928 | 3149 | 68.54 | Israel | 40-50 | A | AQ | Dead Sea | H | Chemoorganotrophic | (55) |
| *Halobellus clavatus* strain CGMCC 1.10118 | 660517 | 3679 | 64.11 | China | 25-50 | A | AQ | Marine solar saltern | H | Heterotrophic | (56) |
| *Halobiforma lacisalsi* AJ5 | 358396 | 4151 | 56.41 | China | 42-45 | FA | AQ | Ayakekum salt lake | H | Chemoorganotrophic | (57) |
| *Halobiforma nitratireducens* JCM 10879 | 1227454 | 3534 | 63.70 | Egypt | 42 | A | S | Soil | H | Chemoorganotrophic | (58) |
| *Halococcus hamelinensis* 100A6 | 1132509 | 3400 | 65.50 | Australia | 37 | A | AQ | Stromatolites in Hamelin pool | H | - | (59) |
| *Halococcus morrhuae* DSM 1307 | 931277 | 3035 | 63.80 | Czech | 30 | A | - | - | H | - | (60) |
| *Halococcus saccharolyticus* DSM 5350 | 1227455 | 3441 | 64.00 | Spain | - | A | AQ | Ponds of salterns | H | Chemoorganotrophic | (61) |
| *Haloferax larsenii* JCM 13917 | 1227460 | 3630 | 62.00 | China | 22-55 | - | AQ | Solar salterns | H | Chemoorganotrophic | (62) |
| *Haloferax mediterranei* ATCC 33500 | 523841 | 3826 | 60.26 | Spain | - | FA | AQ | Seawater evaporation ponds | H | - | (63) |
| *Haloferax sp.* SB29 | 1544718 | 3440 | 60.85 | Saudi Arabia | - | - | AQ | Brine seawater interface | H | - | - |
| *Haloferax volcanii* DS2 | 309800 | 3996 | 65.46 | Israel | 30-40 | A | AQ | Bottom sediments | H | Organotrophic | (64) |
| *Halogeometricum borinquense* DSM 11551 | 469382 | 3894 | 59.97 | Puerto Rico | 40 | A | S | Solar salterns | H | Chemoorganotrophic | (65) |
| *Halogeometricum rufum* strain CGMCC 1.7736 | 553469 | 4155 | 67.65 | China | 25-55 | A | AQ | Marine solar saltern | H | Chemoorganotrophic | (66) |
| *Halogranum amylolyticum* strain CGMCC 1.10121 | 660520 | 5247 | 62.86 | China | 20-50 | A | AQ | Marine solar saltern | H | Chemoorganotrophic | (67) |
| *Halogranum gelatinilyticum* strain CGMCC 1.10119 | 660521 | 3752 | 65.14 | China | 20-50 | A | AQ | Marine solar saltern | H | Chemoorganotrophic | (67) |
| *Halogranum salarium* B-1 | 1210908 | 4824 | 62.20 | South Korea | 37 | A | S | Sea salt | H | Chemoorganotrophic | (68) |
| *Halohasta litchfieldiae* strain DSM 22187 | 1073996 | 3425 | 58.80 | Antarctica | 25-40 | A | AQ | Deep lake | H | Chemoorganotrophic | (69) |
| *Halolamina pelagica* strain CDK2 | 699431 | 3464 | 67.60 | India | 37 | A | S | Natural Saltern | H | Oligotrophic | (70) |
| *Halolamina sp.* CBA1230 strain CBA1230 | 1853690 | 3387 | 66.76 | South Korea | - | - | S | Solar salt | H | - | - |
| *Halomicrobium mukohataei* DSM 12286 | 485914 | 3343 | 65.51 | Argentina | 45 | FA | S | Salt flats | H | - | (71) |
| *Halomicrobium zhouii* strain CGMCC 1.10457 | 767519 | 4091 | 66.20 | China | 20-55 | A | AQ | Marine solar saltern | H | Oligotrophic | (72) |
| *Halonotius sp.* J07HN4 | 1070774 | 3224 | 61.00 | Australia | - | - | AQ | Filtered surface water | H | - | - |
| *Halopelagius inordinatus* strain CGMCC 1.7739 | 553467 | 3559 | 65.02 | China | 20-50 | A | AQ | Marine solar saltern | H | Chemoorganotrophic | (73) |
| *Halopelagius longus* strain CGMCC 1.12397 | 1236180 | 3802 | 65.72 | China | 20-55 | A | S | Salt mine | H | Chemoorganotrophic | (74) |
| *Halopenitus malekzadehii* strain IBRC-M10418 | 1267564 | 3119 | 64.48 | Iran | 30-50 | AN | AQ | Salt lake | H | - | (75) |
| *Halopenitus persicus* strain DC30, IBRC 10041, KCTC 4046 | 1048396 | 3343 | 65.48 | Iran | 25-50 | A | AQ | Salt lake | H | - | (76) |
| *Halophilic archaeon* DL31 | 756883 | 3413 | 62.37 | - | - | - | - | - | H | - | - |
| *Halophilic archaeon* J07HB67 | 1085029 | 2831 | 67.20 | Australia | - | - | AQ | Filtered surface water | H | - | - |
| *Halophilic archaeon* J07HX5 | 1325472 | 2128 | 60.70 | Australia | - | - | AQ | Filtered surface water | H | - | - |
| *Halophilic archaeon* J07HX64 | 1085028 | 3025 | 64.00 | Australia | - | - | AQ | Filtered surface water | H | - | - |
| *Halopiger salifodinae* KCY07-B2 | 1202768 | 4090 | 65.41 | China | 25-50 | A | S | Salt mine | H | - | (77) |
| *Halopiger xanaduensis* SH-6 | 797210 | 4221 | 65.20 | China | 28-45 | A | AQ | Lake sediment | H | - | (78) |
| *Haloquadratum sp.* J07HQX50 | 1238426 | 2865 | 50.70 | Australia | - | - | AQ | Filtered surface water | H | - | - |
| *Haloquadratum walsbyi DSM 16790* | 362976 | 2558 | 47.90 | Egypt | - | A | AQ | Brine pool | H | Phototrophic | (79) |
| *Haloquadratum walsbyi* J07HQW1 | 1238424 | 3574 | 49.40 | Australia | - | A | AQ | Filtered surface water | H | Heterotrophic/Phototrophic | (80) |
| *Haloquadratum walsbyi* J07HQW2 | 1238425 | 3847 | 48.00 | Australia | - | A | AQ | Filtered surface water | H | Heterotrophic/Phototrophic | (80) |
| *Halorhabdus tiamatea* SARL4B | 1033806 | 2988 | 62.77 | Red sea | 15-55 | AN | AQ | Deep see basin | H | - | (81) |
| *Halorientalis regularis* strain IBRC-M 10760 | 660518 | 4204 | 64.95 | China | 20-50 | A | AQ | Marine solar saltern | H | Chemoorganotrophic | (56) |
| *Halorientalis sp.* IM1011 strain IM1011 | 1932360 | 3726 | 65.58 | China | - | - | AQ | Saltern | H | - | - |
| *Halorubrum aidingense* JCM 13560 | 1230454 | 2981 | 67.20 | China | 40-42 | A | AQ | Salt lake | H | Chemoorganotrophic | (82) |
| *Halorubrum distributum* strain E8 | 29283 | 2492 | 66.35 | Iran | - | FA | AQ | Endorheic salt lake | H | - | - |
| *Halorubrum halodurans* strain Cb34 | 1383851 | 3137 | 67.64 | Iran | 20-45 | FA | AQ | Endorheic salt lake | H | Chemoorganotrophic | (83) |
| *Halorubrum hochstenium* ATCC 700873 | 1227481 | 2931 | 69.10 | - | - | - | - | - | H | - | - |
| *Halorubrum lacusprofundi* ATCC 49239 | 416348 | 3495 | 63.95 | Antarctica | - | A | AQ | Deep Lake | H | Chemoorganotrophic | (84) |
| *Halorubrum lipolyticum* DSM 21995 | 1227482 | 3220 | 68.00 | China | 25-58 | A | AQ | Salt lake | H | Chemoorganotrophic | (82) |
| *Halorubrum persicum* strain C49 | 1383844 | 3256 | 65.88 | Iran | 20-45 | FA | AQ | Endorheic salt lake | H | Chemoorganotrophic | (85) |
| *Halorubrum saccharovorum* strain H3 | 2248 | 2334 | 65.70 | Russia | - | - | AQ | Salt lake sediments | H | - | - |
| *Halorubrum sp.* AJ67 | 1173487 | 3364 | 58.70 | Argentina | - | A | AQ | Shallow lake | H | Chemoorganotrophic | (86) |
| *Halorubrum sp.* E3 | 1483400 | 4984 | 65.52 | Iran | - | FA | AQ | Endorheic salt lake | H | - | - |
| *Halorubrum sp.* Ib24 | 1383850 | 2609 | 67.53 | Iran | - | FA | AQ | Endorheic salt lake | H | - | - |
| *Halorubrum sp.* J07HR59 | 1238428 | 1832 | 59.50 | Australia | - | - | AQ | Filtered surface water | H | - | - |
| *Halorubrum sp.* SD683 strain SD683 | 1855873 | 3030 | 69.00 | Namibia | - | - | AQ | Solar saltern surface water | H | - | - |
| *Halorubrum sp.* WN019 | 2032630 | 3214 | 67.4 | China | - | - | S | Saline alkaline soil | H | - | - |
| *Halorubrum vacuolatum* strain DSM 8800 | 63740 | 3371 | 62.89 | Kenya | - | - | AQ | Lake Magadi | H | - | - |
| *Halosarcina pallida* JCM 14848 | 1227487 | 4246 | 65.60 | United States | 25-45 | - | AQ | Sulfide-rich spring | H | Chemoorganotrophic | (87) |
| *Halosimplex carlsbadense* 2-9-1 | 797114 | 4458 | 67.70 | United States | 22-50 | A | S | Salt crystal | H | - | (88) |
| *Halostagnicola larsenii* XH-48 | 797299 | 4086 | 60.87 | China | 25-50 | A | AQ | Saline lake sediment | H | - | (89) |
| *Halostagnicola sp.* A56 | 1495067 | 2711 | 60.50 | India | - | A | S | Andaman Islands | H | - | (90) |
| *Haloterrigena daqingensis strain* JX313 | 588898 | 3795 | 61.35 | China | 20-50 | A | S | Saline-alkaline field | H | Chemoorganotrophic | (91) |
| *Haloterrigena limicola* JCM 13563 | 1230457 | 3519 | 61.80 | China | 30-61 | A | AQ | Salt lake | H | Chemoorganotrophic | (92) |
| *Haloterrigena turkmenica* DSM 5511 | 543526 | 5113 | 64.25 | Turkey | 29-57 | A | AQ | Salt lake | H | Chemoorganotrophic | (93) |
| *Halovenus aranensis* strain IBRC-M10015 | 890420 | 3354 | 61.02 | Iran | 25-50 | A | AQ | Salt lake | H | Chemoorganotrophic | (94) |
| *Halovivax ruber* XH-70 | 797302 | 3099 | 64.30 | China | 25-45 | A | AQ | Saline lake | H | - | (95) |
| *Hyperthermus butylicus* DSM 5456 | 415426 | 1602 | 53.70 | Portugal | 80-108 | AN | AQ | Sea floor of a solfataric environment | T | - | (96) |
| *Ignicoccus hospitalis* KIN4/I | 453591 | 1434 | 56.50 | Iceland | 73-98 | AN | AQ | Hydrothermal vent system | T | Chemolithotrophic | (97) |
| *Ignicoccus islandicus* DSM 13165 | 940295 | 1510 | 44.90 | Atlantic Ocean | 70-98 | AN | AQ | Submarine hypothermal system | T | Chemolithoautotrophic | (98) |
| *Ignisphaera aggregans* DSM 17230 | 583356 | 1929 | 35.70 | New Zealand | 95 | AN | AQ | Hot sprint | T | Autotrophic | (99) |
| *Lokiarchaeum sp.* GC14_75 | 1538547 | 5378 | 31.09 | Arctic Ocean | - | AN | AQ | Hydrothermal vent sediment | - | - | - |
| Marine Group I *thaumarchaeote* SCGC AAA799-B03 | 1502289 | 1339 | 33.70 | Red sea | - | A | AQ | Brine and seawater interface | H | - | - |
| Marine Group I *thaumarchaeote* SCGC AAA799-P11 | 1502295 | 1432 | 33.67 | Red sea | - | A | AQ | Brine and seawater interface | H | - | - |
| Marine Group II *euryarchaeote* MED-G34 | 2007295 | 899 | 53.64 | Spain | - | A | AQ | Marine water | - | Heterotrophic | (100) |
| Marine Group II *euryarchaeote* MED-G36 | 2007297 | 828 | 43.65 | Spain | - | A | AQ | Marine water | - | Heterotrophic | (100) |
| *Metallosphaera sedula* DSM 5348 | 399549 | 2256 | 46.20 | Italy | 70 | A | S | Volcanic Field | TA | Mixotrophic | (101) |
| *Metallosphaera yellowstonensis* MK1 | 671065 | 3340 | 47.70 | United States | - | A | AQ | Fresh water hot spring | TA | Chemotrophic | (102) |
| *Methanobacterium congolense* strain Buetzberg | 118062 | 2340 | 36.05 | - | 6-50 | AN | AQ | Biogas sludge | M | Hydrogenotrophic | (103) |
| *Methanobacterium lacus* strain AL-21 | 877455 | 2493 | 35.80 | France | 14-41 | AN | AQ | Lake profundal sediment | M | Hydrogenotrophic | (104) |
| *Methanobacterium paludis* strain SWAN-1 | 868131 | 2394 | 35.70 | United States | 32-37 | AN | S | Peatland | M | Hydrogenotrophic | (104) |
| *Methanobacterium sp.* A39 | 1860100 | 3065 | 33.24 | Australia | - | - | - | - | M | - | - |
| *Methanobacterium sp.* MB1 | 1379702 | 2013 | 39.70 | Germany | - | AN | S | Rural biogas plant | M | Hydrogenotrophic | (105) |
| *Methanobrevibacter arboriphilus* JCM 13429 = DSM 1125 | 1300164 | 1961 | 25.43 | - | - | - | S | Wetwood of methane-emitting trees | M | Autotrophic | - |
| *Methanobrevibacter curvatus* strain DSM 11111 | 49547 | 1968 | 25.72 | - | - | - | Ho | Hindguts | M | - | (106) |
| *Methanobrevibacter cuticularis* strain DSM 11139 | 47311 | 2061 | 26.79 | - | - | - | Ho | Hindguts | M | - | (106) |
| *Methanobrevibacter filiformis* strain DSM 11501 | 55758 | 1920 | 26.99 | - | - | - | Ho | Hindguts | M | - | (106) |
| *Methanobrevibacter millerae* strain SM9 | 230361 | 2260 | 31.81 | New Zealand | - | AN | Ho | Rumen fluid | M | Hydrogenotrophic | (107) |
| *Methanobrevibacter oralis* strain DSM 7256 | 66851 | 1993 | 27.71 | - | - | - | Ho | Oral cavity | M | - | (106) |
| *Methanobrevibacter ruminantium* M1 | 634498 | 2209 | 32.60 | United States | - | AN | Ho | Rumen | M | Hydrogenotrophic | (108) |
| *Methanobrevibacter smithii* ATCC 35061 | 420247 | 1783 | 31.00 | United States | 37-40 | AN | S | Sewage digester | M | Syntrophic | (109) |
| *Methanobrevibacter sp.* 87.7 strain 87.7 | 387957 | 1573 | 24.41 | France | - | AN | Ho | Rumen | M | - | - |
| *Methanobrevibacter sp.* A27 | 1860099 | 1728 | 30.06 | Australia | - | - | - | - | M | - | - |
| *Methanobrevibacter sp.* AbM4 | 224719 | 1663 | 29.00 | - | - | - | Ho | Ovine rumen | M | - | - |
| *Methanobrevibacter sp.* YE315 | 1609968 | 1942 | 34.25 | Australia | - | - | Ho | Pooled rumen fluid | M | - | - |
| *Methanocaldococcus infernus* ME | 573063 | 1439 | 33.60 | Atlantic Ocean | 85 | AN | AQ | Deep-sea hydrothermal chimneys | M | Lithotrophic | (110) |
| *Methanocaldococcus jannaschii* DSM 2661 | 243232 | 1787 | 31.27 | Mexico | 85 | AN | AQ | Hydrothermal vent | T | Autotrophic | (111) |
| *Methanocella arvoryzae* MRE50 | 351160 | 3071 | 54.60 | Italy | 15-60 | - | S | Rhizome | M | Hydrogenotrophic | (112) |
| *Methanocella paludicola* SANAE | 304371 | 2993 | 54.90 | Japan | 37 | - | S | Rice paddy samples | M | Syntrophic | (113) |
| *Methanococcoides burtonii* DSM 6242 | 259564 | 2242 | 40.80 | Antarctica | 23.4 | AN | AQ | Bottom of Ace lake | PP | Methylotrophic | (114) |
| *Methanococcus aeolicus* Nankai-3 | 419665 | 1490 | 30.00 | Japan | 46 | AN | AQ | Shallow and deep marine sediments | M | Autotrophic | (115) |
| *Methanococcus maripaludis* S2 | 267377 | 1722 | 33.10 | United States | 35-40 | AN | AQ | Salt marsh sediment | M | Hydrogenotrophic | (116) |
| *Methanococcus voltae* A3 | 456320 | 1658 | 28.60 | United States | 30-37 | AN | AQ | Salt marsh | M | Autotrophic | (117) |
| *Methanocorpusculum labreanum* Z | 410358 | 1739 | 50.00 | United States | 37 | AN | AQ | Surface sediment | M | - | (118) |
| *Methanoculleus bourgensis* MS2 | 1201294 | 2575 | 60.64 | France | - | AN | AQ | Sewage sludge digester | M | Hydrogenotrophic | (119) |
| *Methanoculleus marisnigri* JR1 | 368407 | 2476 | 62.10 | Black Sea | 21-25 | AN | AQ | Anoxic sediment | M | - | (120) |
| *Methanofollis liminatans* DSM 4140 | 28892 | 2423 | 60.96 | Germany | 37-40 | AN | AQ | Industrial wastewater | - | Hydrogenotrophic | (121) |
| *Methanogenic archaeon* ISO4-H5 | 1495144 | 1805 | 54.00 | New Zealand | 39 | AN | Ho | Ovine rumen | M | Methylotrophic | (122) |
| *Methanohalobium evestigatum* Z-7303 | 644295 | 2250 | 36.40 | Russia | 50 | AN | AQ | Mud from a salt lagoon | M | - | - |
| *Methanohalophilus mahii* DSM 5219 | 547558 | 1986 | 42.60 | United States | - | AN | AQ | Great salt lake | M | Hydrogenotrophic | (123) |
| *Methanolobus profundi* strain Mob M | 487685 | 2902 | 42.57 | Japan | 30 | AN | AQ | Deep subsurface sediments | M | Methylotrophic | (124) |
| *Methanolobus psychrophilus* R15 | 1094980 | 3132 | 44.60 | China | 4-18 | AN | AQ | Freshwater wetland | PP | Methylotrophic | (125) |
| *Methanolobus sp.* T82-4 | 1794908 | 2692 | 43.69 | United States | - | - | S | Hydraulically fractured well fluid | M | - | - |
| *Methanolobus tindarius* DSM 2278 | 1090322 | 2912 | 39.80 | Italy | 10-45 | - | S | Coastal sediments | M | - | (126) |
| *Methanomassiliicoccales archaeon* RumEn M1 | 1713724 | 1877 | 62.09 | Austria | - | - | Ho | Rumen fluid | M | - | - |
| *Methanomassiliicoccales archaeon* RumEn M2 | 1713725 | 1175 | 54.60 | Austria | - | - | Ho | Rumen fluid | M | - | - |
| *Methanomethylovorans hollandica* DSM 15978 | 867904 | 2551 | 35.42 | Netherlands | 12-40 | AN | AQ | Eutrophic freshwater pond | M | Methylotrophic | (127) |
| *Methanomicrobiales archaeon* 53_19 | 1641394 | 2860 | 52.46 | United States | - | - | S | Oil reservoir | M | Hydrogenotrophic | - |
| *Methanomicrobiales archaeon* HGW-Methanomicrobiales-1 | 2013817 | 2456 | 51.44 | Japan | - | - | AQ | groundwater | M | - | - |
| *Methanomicrobiales archaeon* HGW-Methanomicrobiales-2 | 2013818 | 2624 | 60.8 | Japan | - | - | AQ | groundwater | M | - | - |
| *Methanomicrobiales archaeon* HGW-Methanomicrobiales-4 | 2013820 | 2087 | 46.2 | Japan | - | - | AQ | groundwater | M | - | - |
| *Methanomicrobiales archaeon* HGW-Methanomicrobiales-6 | 2013822 | 2528 | 61.48 | Japan | - | - | AQ | groundwater | M | - | - |
| *Methanoplanus limicola* DSM 2279 | 937775 | 2928 | 42.19 | Italy | 17-41 | AN | AQ | Mud of drilling swamp | M | Chemoorganotrophic | (128) |
| *Methanoplanus petrolearius* DSM 11571 | 679926 | 2779 | 47.40 | Gulf of Guinea | 25-45 | AN | AQ | Offshore oil field | M | Methylotrophic | (129) |
| *Methanopyrus kandleri* AV19 | 190192 | 1687 | 61.20 | Gulf of California | 98 | AN | AQ | Guaymas hot vents | T | Chemoautolithotrophic | (130) |
| *Methanoregula boonei* 6A8 | 456442 | 2450 | 54.50 | United States | 37 | AN | S | Sour digester | AC | - | (131) |
| *Methanoregula formicica* SMSP | 593750 | 2815 | 55.20 | Japan | 10-40 | AN | AQ | Granular sludge | M | Hydrogenotrophic | (132) |
| *Methanosaeta concilii* GP6 | 990316 | 2791 | 50.96 | Japan | 35-40 | AN | AQ | Pear waste fermenter | M | - | (133) |
| *Methanosaeta harundinacea* 6Ac | 1110509 | 2358 | 53.97 | China | 25-45 | AN | S | Sludge blanket (UASB) reactors | M | - | (134) |
| *Methanosaeta thermophila* PT | 349307 | 1673 | 53.50 | - | 55-60 | AN | AQ | Digester sludge | M | Acetotrophic | (135) |
| *Methanosalsum zhilinae* DSM 4017 | 679901 | 1972 | 39.20 | Egypt | 44-50 | AN | AQ | Sediment of alkaline lake | H | Lithoheterotrophic | (136) |
| *Methanosarcina acetivorans* C2A | 188937 | 4468 | 42.70 | United States | 35-40 | AN | AQ | Marine sediment | M | Carboxidotrophic | (137) |
| *Methanosarcina barkeri* 3 | 1434107 | 3460 | 39.08 | - | - | - | - | - | M | - | - |
| *Methanosarcina barkeri* str. Fusaro | 269797 | 3616 | 39.26 | Italy | 35-40 | AN | AQ | Freshwater lake | M | Methylotrophic | (138) |
| *Methanosarcina horonobensis* HB-1 = JCM 15518 | 1434110 | 4070 | 41.31 | Japan | 20-42 | AN | AQ | Groundwater | M | - | (139) |
| *Methanosarcina lacustris* Z-7289 | 1434111 | 3201 | 41.82 | Switzerland | 1-35 | AN | AQ | Anoxic lake sediment | M | - | (140) |
| *Methanosarcina mazei* Go1 | 192952 | 3303 | 41.50 | Germany | 30-40 | AN | AQ | Sewage digester | M | - | (141) |
| *Methanosarcina sp.* 2.H.A.1B.4 | 1483600 | 3154 | 42.93 | Columbia | - | - | AQ | River sediment | M | - | - |
| *Methanosarcina sp.* Ant1 | 1882735 | 3225 | 40.10 | Antarctica | - | - | S | Soil | M | - | - |
| *Methanosarcina sp.* E03.2 | 1715806 | 2654 | 41.30 | Germany | 45 | A | S | AN digester sludge | M | - | - |
| *Methanosarcina sp.* MTP4 | 1434100 | 3238 | 45.93 | - | - | - | - | - | M | - | - |
| *Methanosarcina sp.* WH1 | 1434102 | 3152 | 41.82 | - | - | - | - | - | M | - | - |
| *Methanosphaera cuniculi* strain 1R-7 | 1077256 | 1593 | 28.03 | Italy | 35-40 | AN | Ho | Intestine | M | - | (142) |
| *Methanosphaera sp.* WGK6 | 1561964 | 1456 | 27.70 | Australia | - | - | Ho | Forestomach | M | Autotrophic | (143) |
| *Methanosphaera stadtmanae* DSM 3091 | 339860 | 1533 | 27.60 | Germany | 36-40 | AN | Ho | Human feces | M | Autotrophic | (144) |
| *Methanosphaerula palustris* E1-9c | 521011 | 2655 | 55.40 | United States | 30 | AN | AQ | Organically rich wetland | M | Hydrogenotrophic | (145) |
| *Methanospirillum hungatei* JF-1 | 323259 | 3087 | 45.10 | United States | 37 | AN | AQ | Sewage sludge | M | Syntrophic | (146) |
| *Methanothermobacter thermautotrophicus* str. Delta H | 187420 | 1868 | 49.50 | United States | 65-70 | AN | AQ | Sewage sludge | M | Autotrophic | (147) |
| *Methanothermus fervidus* DSM 2088 | 523846 | 1283 | 31.60 | Iceland | 80-88 | AN | AQ | Hot solfataric spring | T | Chemolithoautotrophic | (148) |
| *Methanotorris igneus* Kol 5 | 880724 | 1753 | 32.30 | Iceland | - | - | AQ | Shallow submarine vent system | M | - | - |
| *miscellaneous Crenarchaeota* group archaeon SMTZ-80 | 1685135 | 1369 | 34.45 | United States | - | AN | AQ | Sulphate-methane transition zone estuary sediments | - | - | - |
| *miscellaneous Crenarchaeota* group archaeon SMTZ1-55 | 1685133 | 1122 | 54.96 | United States | - | AN | AQ | Sulphate-methane transition zone estuary sediments | - | - | - |
| *miscellaneous Crenarchaeota* group-1 archaeon SG8-32-1 | 1685124 | 1116 | 36.66 | United States | - | AN | AQ | Sulphate-rich zone estuary sediments | - | - | - |
| *miscellaneous Crenarchaeota* group-1 archaeon SG8-32-3 | 1685125 | 640 | 44.01 | United States | - | AN | AQ | Sulphate-rich zone estuary sediments | - | - | - |
| *miscellaneous Crenarchaeota* group-15 archaeon DG-45 | 1685127 | 1314 | 62.09 | United States | - | AN | AQ | Sulphate-rich zone estuary sediments | - | - | - |
| *miscellaneous Crenarchaeota* group-6 archaeon AD8-1 | 1685126 | 1505 | 32.43 | United States | - | AN | AQ | Sulphate-rich zone estuary sediments | - | - | - |
| *Nanoarchaeota archaeon* strain 1420 | 2026764 | 609 | 36.3 | - | - | - | AQ | Marine water sample | - | - | - |
| *Nanoarchaeote Nst1* | 1294122 | 647 | 24.30 | United States | 60-90 | AN | AQ | Hot spring water and gravel | TA | - | (149) |
| *Nanoarchaeum equitans* Kin4-M | 228908 | 536 | 31.60 | - | - | AN | AQ | Submarine hot vent | T | Parasitic | (150) |
| *Nanosalina sp.* (strain J07AB43) (Strain: J07AB43) | 889948 | 1673 | 43.60 | Australia | - | - | AQ | Lake surface water | H | - | - |
| *Nanosalinarum sp.* (strain J07AB56) | 889962 | 1402 | 55.40 | Australia | - | - | AQ | Lake surface water | H | - | - |
| *Natrialba asiatica DSM* 12278 | 29540 | 4175 | 62.40 | - | - | - | AQ | Solar salt and beach sand | H | - | - |
| *Natrialba magadii* ATCC 43099 | 547559 | 4203 | 61.03 | Kenya | - | A | S | Lake Magadi | H | - | - |
| *Natrinema ejinorense* strain JCM 13890 | 373386 | 4191 | 63.87 | China | 25-50 | A | AQ | Saline lake | H | Chemoorganotrophic | (151) |
| *Natrinema pellirubrum* DSM 15624 | 797303 | 4138 | 57.01 | Italy | 20-45 | A | S | Salted hides or spoiled fish | H | Chemoorganotrophic | (152) |
| *Natrinema salaciae* strain DSM 25055 | 1186196 | 4688 | 64.92 | Mediterranean Sea | 22-25 | FA | AQ | Anoxic lake | H | - | (153) |
| *Natrinema sp.* CBA1119 | 1608465 | 4602 | 62.27 | South Korea | - | - | S | Solar salt | H | - | - |
| *Natrinema sp.* J7-2 | 406552 | 4296 | 64.06 | China | - | - | S | Salt mine | H | - | (154) |
| *Natrinema versiforme* JCM 10478 | 1227496 | 4160 | 64.00 | China | 4-60 | AN | AQ | Salt lake | H | Chemoorganotrophic | (155) |
| *Natronoarchaeum philippinense* strain DSM 27208 | 558529 | 3284 | 65.16 | Japan | 20-50 | A | AQ | Marine solar saltern | H | - | (156) |
| *Natronobacterium gregoryi* SP2 | 797304 | 3624 | 62.2 | Kenya | - | - | AQ | Water | H | - | - |
| *Natronobacterium texcoconense* strain DSM 24767 | 1095778 | 4046 | 62.87 | Mexico | 25-45 | A | S | Soil | H | - | (157) |
| *Natronococcus occultus* SP4 | 694430 | 4153 | 54.84 | Kenya | 37 | A | AQ | Lake Magadi | H | - | - |
| *Natronolimnobius baerhuensis* strain CGMCC 1.3597 | 253108 | 3662 | 60.20 | China | 37-45 | A | AQ | Soda lake | H | Chemoorganotrophic | (158) |
| *Natronolimnobius innermongolicus* JCM 12255 | 1227499 | 4400 | 64.30 | Mexico | 19-54 | A | AQ | Soda lake | H | Chemoorganotrophic | (158) |
| *Natronomonas moolapensis* 8.8.11 | 268739 | 2723 | 64.50 | Australia | 37-40 | A | S | Marine solar saltern crystallizer | H | Chemoorganotrophic | (159) |
| *Natronomonas pharaonis* DSM 2160 | 348780 | 2764 | 63.08 | Egypt | - | A | AQ | Lake Gabara | H | - | (160) |
| *Natronorubrum sulfidifaciens* JCM 14089 | 1230460 | 3428 | 61.80 | China | 20-55 | A | AQ | Salt lake | H | Chemoorganotrophic | (161) |
| *Natronorubrum tibetense* GA33 | 1114856 | 4671 | 62.30 | China | 45 | - | AQ | Soda lake | H | Chemoorganotrophic | - |
| *Nitrosopumilus maritimus* SCM1 | 436308 | 1795 | 34.20 | United States | 9-29 | A | AQ | Salt water aquarium | - | Chemolithotrophic | (162) |
| *Nitrosopumilus sp.* PRT-SC01 | 1527301 | 709 | 33.13 | Puerto Rico | - | - | AQ | Oceanic Trench | AO | Chemoautotrophical | - |
| *Nitrososphaera viennensis* EN76 | 926571 | 3117 | 52.70 | Austria | 42 | A | S | Soil | AO | Mixotrophic | (163) |
| *Nitrosotalea sp.* CS strain NCS1 | 1903276 | 2462 | 37.46 | - | - | - | - | - | - | - | - |
| *Nitrosotalea sp.* Nd2 strain NSIN | 1499975 | 1881 | 37.37 | - | - | - | - | - | AO | Chemoautotrophical | - |
| *Palaeococcus pacificus* DY20341 | 1343739 | 1991 | 43.00 | Pacific Ocean | 50-90 | AN | AQ | Deep sea hydrothermal sediments | T | Chemoorganoheterotrophic | (164) |
| *Picrophilus torridus* DSM 9790 | 263820 | 1535 | 36.00 | Japan | 60 | A | AQ | Hot spring | T | Heterotrophic | (165) |
| *Pyrobaculum aerophilum* str. IM2 | 178306 | 2590 | 51.40 | Italy | 100 | FA | AQ | Boiling marine water hole | T | Lithotrophic | (166) |
| *Pyrobaculum arsenaticum* DSM 13514 | 340102 | 2296 | 55.10 | Italy | - | AN | AQ | Hot spring | T | Chemoautotrophic | (167) |
| *Pyrococcus furiosus* DSM 3638 | 186497 | 2045 | 40.80 | Italy | 100 | AN | AQ | Shallow marine solfatara | T | Heterotrophic | (168) |
| *Pyrococcus horikoshii* OT3 | 70601 | 2077 | 41.90 | Japan | 98 | AN | AQ | Hydrothermal vent site | T | Heterotrophic | (169) |
| *Pyrococcus yayanosii* CH1 | 529709 | 1865 | 51.60 | Atlantic Ocean | 80-180 | AN | AQ | Hydrothermal vent | PZ | Heterotrophic | (170) |
| *Pyrodictium delaneyi* strain Su06 | 1273541 | 2035 | 53.91 | Pacific Ocean | 90 | AN | AQ | Active hydrothermal chimney | T | Chemolithoautotrophic | (171) |
| *Pyrodictium occultum* strain PL-19 | 2309 | 1602 | 63.50 | Italy | 105 | AN | AQ | Marine hydrothermal sediment | T | - | - |
| *Pyrolobus fumarii* 1A | 694429 | 1967 | 54.90 | Atlantic Ocean | 106 | FA | AQ | Hydrothermal vent | T | Chemolithoautotrophic | (172) |
| *Salinarchaeum sp.* Harcht-Bsk1 | 1333523 | 3013 | 66.60 | Russia | 40 | A | AQ | Hypersaline lake sediment | H | Organotrophic | (173) |
| *Staphylothermus marinus* F1 | 399550 | 1570 | 35.70 | Italy | 92 | AN | AQ | Geothermally heated sediments | T | Heterotrophic | (174) |
| *Sulfolobales archaeon* AZ1 | 1326980 | 1975 | 47.00 | Mexico | 65 |  | AQ | Hot springs | TA | Chemotrophic | (175) |
| *Sulfolobus acidocaldarius* DSM 639 | 330779 | 2221 | 36.70 | United States | 70-75 | A | AQ | Acidic hot spring | TA | - | (176) |
| *Sulfolobus solfataricus* P2 | 273057 | 2938 | 35.80 | Italy | 85 | A | AQ | Hot spring | T | Heterotrophic | (177) |
| *Sulfolobus sp.* A20 | 1891280 | 2494 | 34.78 | Costa Rica | 65-85 | A | AQ | Hot spring | T | Mixotrophic | (178) |
| *Sulfolobus tokodaii* str. 7 | 273063 | 2805 | 32.80 | Japan | 80 | A | AQ | Hot springs | T | - | (179) |
| *Thaumarchaeota archaeon* MY3 | 1353260 | 3684 | 34.06 | United States | 25-30 | A | S | Coal tar contaminated soil | AO | Chemolithoautotrophic | (180) |
| *Thaumarchaeota archaeon* N4 | 1407055 | 1946 | 42.20 | - | - | - | - | - | AO | - | - |
| *Thaumarchaeota archaeon* SCGC AB-539-E09 | 1198115 | 646 | 43.30 | Denmark | - | AN | AQ | Oceanic sediment | AO | - | - |
| *Thaumarchaeota archaeon* strain SAT137 | 2026795 | 2070 | 36.11 | - | - | - | AQ | Marine water sample | - | - | - |
| *Thermococcales archaeon* 44_46 | 1635283 | 1983 | 43.45 | United States | - | - | S | Oil reservoir | T | - | - |
| *Thermococcus celer* strain Vu 13 | 2264 | 1903 | 56.45 | Italy | - | - | AQ | Hydrothermal fluid | T | - | - |
| *Thermococcus celericrescens* strain DSM 17994 | 227598 | 2330 | 54.29 | Japan | 50-85 | AN | AQ | Hydrothermal vent | T | Chemoorganotrophic | (181) |
| *Thermococcus chitonophagus* strain 1 | 54262 | 2111 | 44.92 | Mexican | 60-93 | AN | AQ | Hydrothermal vent site | T | Organotrophic | (182) |
| *Thermococcus gammatolerans* EJ3 | 593117 | 2157 | 53.60 | United States | 88 | AN | AQ | Hydrothermal vent | T | Organotrophic | (183) |
| *Thermococcus kodakarensis* KOD1 | 69014 | 2301 | 52.00 | Japan | 85 | AN | S | Solfatara | T | Heterotrophic | (184) |
| *Thermococcus onnurineus* NA1 | 523850 | 1976 | 51.30 | Pacific Ocean | 80 | AN | AQ | Deep sea hydrothermal vent | T | Heterotrophic/Carboxydotrophic | (185) |
| *Thermococcus pacificus* strain P-4 | 71998 | 1872 | 54.24 | New Zealand | 80-88 | AN | AQ | Hydrothermal sediment | T | Heterotrophic | (186) |
| *Thermococcus sibiricus* MM 739 | 604354 | 2035 | 40.20 | Russia | 40-88 | AN | S | High-temperature Samotlor oil reservoir | T | Organotrophic | (187) |
| *Thermococcus sp.* 4557 | 1042877 | 2133 | 56.10 | United States | - | AN | AQ | Hydrothermal vent | T | - | - |
| *Thermococcus sp.* 5-4 strain 5-4 | 2008440 | 1961 | 55.69 | - | - | - | AQ | Black smoker | T | - | - |
| *Thermococcus sp.* ES1 | 582419 | 2014 | 40.30 | Pacific Ocean | 82 | AN | AQ | Hydrothermal vent | T | Heterotrophic | (188) |
| *Thermofilum pendens* Hrk 5 | 368408 | 1876 | 57.68 | Iceland | 88 | AN | AQ | Hot spring | T | - | - |
| *Thermofilum sp.* 1807-2 | 1550241 | 1454 | 47.94 | Russia | 70-90 | AN | AQ | Hot spring sediments | T | Organotrophic | (189) |
| *Thermofilum sp.* 1910b | 1365176 | 1896 | 46.50 | Russia | 70-90 | AN | AQ | Hot spring sediments | T | Chemoorganotrophic | (189) |
| *Thermofilum sp.* ex4484_79 | 2012527 | 1716 | 38.47 | United States | - | - | AQ | Deep-sea hydrothermal vent sediments | T | - | - |
| *Thermogladius cellulolyticus* 1633 | 1184251 | 1414 | 55.60 | Russia | 84 | AN | AQ | Thermal pool | T | - | (190) |
| *Thermoplasma acidophilum* DSM 1728 | 273075 | 1482 | 46.00 | United States | 59 | FA | S | Burning refuse coal pile | T | - | - |
| *Thermoplasmatales archaeon* A-plasma | 667135 | 2276 | 46.23 | United States | - | - | S | Pyrite surfaces | TA | - | - |
| *Thermoplasmatales archaeon* B_DKE strain B_DKE | 1961136 | 1928 | 44.47 | Germany | - | - | S | Pyrite mine | TA | - | - |
| *Thermoplasmatales archaeon* BRNA1 | 1054217 | 1528 | 58.30 | - | 39 | AN | Ho | Rumen | T | - | - |
| *Thermoplasmatales archaeon* DG-70 | 1803813 | 3483.00 | 41.69 | United States | - | AN | AQ | Methane-rich estuary sediments | TA | - | - |
| *Thermoplasmatales archaeon* DG-70-1 | 1803814 | 4270.00 | 41.44 | United States | - | AN | AQ | Methane-rich estuary sediments | TA | - | - |
| *Thermoplasmatales archaeon* E-plasma | 667137 | 1661 | 38.39 | United States | - | - | S | Pyrite surfaces | TA | - | - |
| *Thermoplasmatales archaeon* Gpl | 261391 | 1917 | 37.91 | United States | - | - | - | - | TA | - | - |
| *Thermoplasmatales archaeon* I-plasma | 667138 | 1693 | 44.30 | United States | - | - | S | Pyrite surfaces | TA | - | - |
| *Thermoplasmatales archaeon* SCGC AB-539-C06 | 1242690 | 780 | 35.10 | Denmark | - | AN | AQ | Marine sediment | T | - | - |
| *Thermoplasmatales archaeon* SCGC AB-539-N05 | 1198116 | 844 | 36.50 | Denmark | - | AN | AQ | Oceanic sediment | T | - | - |
| *Thermoplasmatales archaeon* SCGC AB-540-F20 | 1242866 | 1255 | 35.60 | Denmark | - | AN | AQ | Marine sediment | T | - | - |
| *Thermoplasmatales archaeon* SG8-52-1 | 1803816 | 1877.00 | 31.05 | United States | - | AN | AQ | Sulphate-methane transition zone estuary sediments | TA | - | - |
| *Thermoplasmatales archaeon* SG8-52-3 | 1803818 | 1739.00 | 30.37 | United States | - | AN | AQ | Sulphate-methane transition zone estuary sediments | TA | - | - |
| *Thermoplasmatales archaeon* SM1-50 | 1803815 | 1890.00 | 39.01 | United States | - | AN | AQ | Sulphate-methane transition zone estuary sediments | TA | - | - |
| *Thermoproteus tenax* Kra 1 | 768679 | 2047 | 55.10 | Iceland | 96 |  | AQ | Solfatara | T | Chemolithoautotrophic | (191) |
| *Thermoproteus uzoniensis* 768-20 | 999630 | 2185 | 59.70 | Russia | 74-102 | AN | AQ | Solfataric thermal field | T | Autotrophic | (192) |
| *Thermosphaera aggregans* DSM 11486 | 633148 | 1387 | 46.70 | United States | 65-90 | AN | AQ | Hot solfataric spring | T | Heterotrophic | (193) |
| *Thorarchaeota archaeon* SMTZ-45 | 1706443 | 2257 | 43.16 | United States | - | AN | AQ | Sulphate-methane transition zone estuary sediments | M | - | - |
| *Thorarchaeota archaeon* SMTZ1-45 | 1706444 | 3208 | 42.16 | United States | - | AN | AQ | Sulphate-methane transition zone estuary sediments | M | - | - |
| *Thorarchaeota archaeon* SMTZ1-83 | 1706445 | 3029 | 49.28 | United States | - | AN | AQ | Sulphate-methane transition zone estuary sediments | M | - | - |
| *uncultured archaeon* A07HB70 | 1412872 | 2513 | 65.00 | Australia | - | - | AQ | Hypersaline water | H | - | - |
| *uncultured archaeon* A07HN63 | 1412873 | 2505 | 55.87 | Australia | - | - | AQ | Hypersaline water | H | - | - |
| *uncultured archaeon* A07HR60 | 1412874 | 2853 | 56.67 | Australia | - | - | AQ | Hypersaline water | H | - | - |
| *uncultured archaeon* A07HR67 | 1412871 | 2887 | 62.13 | Australia | - | - | AQ | Hypersaline water | H | - | - |
| *Vulcanisaeta distributa* DSM 14429 | 572478 | 2493 | 45.40 | Japan | 85-90 | AN | AQ | Hot spring | T | Heterotrophic | (194) |

**References**

1. You, X. Y., Liu, C., Wang, S. Y., Jiang, C. Y., Shah, S. A., Prangishvili, D., She, Q., Liu, S. J., and Garrett, R. A. (2011) Genomic analysis of Acidianus hospitalis W1 a host for studying crenarchaeal virus and plasmid life cycles. *Extremophiles* **15**, 487-497

2. He, H., Yang, Y., Xia, J., Ding, J., Zhao, X., and Nie, Z. (2008) Growth and surface properties of new thermoacidophilic Archaea strain Acidianus manzaensis YN-25 grown on different substrates *Transactions of Nonferrous Metals Society of China* **18**, 5

3. Mardanov, A. V., Svetlitchnyi, V. A., Beletsky, A. V., Prokofeva, M. I., Bonch-Osmolovskaya, E. A., Ravin, N. V., and Skryabin, K. G. (2010) The genome sequence of the crenarchaeon Acidilobus saccharovorans supports a new order, Acidilobales, and suggests an important ecological role in terrestrial acidic hot springs. *Appl Environ Microbiol* **76**, 5652-5657

4. Hawkes, R. B., Franzmann, P. D., O'Hara, G., and Plumb, J. J. (2006) Ferroplasma cupricumulans sp. nov., a novel moderately thermophilic, acidophilic archaeon isolated from an industrial-scale chalcocite bioleach heap. *Extremophiles* **10**, 525-530

5. Schouten, S., Baas, M., Hopmans, E. C., Reysenbach, A. L., and Damste, J. S. (2008) Tetraether membrane lipids of Candidatus "Aciduliprofundum boonei", a cultivated obligate thermoacidophilic euryarchaeote from deep-sea hydrothermal vents. *Extremophiles* **12**, 119-124

6. Kawarabayasi, Y., Hino, Y., Horikawa, H., Yamazaki, S., Haikawa, Y., Jin-no, K., Takahashi, M., Sekine, M., Baba, S., Ankai, A., Kosugi, H., Hosoyama, A., Fukui, S., Nagai, Y., Nishijima, K., Nakazawa, H., Takamiya, M., Masuda, S., Funahashi, T., Tanaka, T., Kudoh, Y., Yamazaki, J., Kushida, N., Oguchi, A., Kikuchi, H., and et al. (1999) Complete genome sequence of an aerobic hyper-thermophilic crenarchaeon, Aeropyrum pernix K1. *DNA Res* **6**, 83-101, 145-152

7. Klenk, H. P., Clayton, R. A., Tomb, J. F., White, O., Nelson, K. E., Ketchum, K. A., Dodson, R. J., Gwinn, M., Hickey, E. K., Peterson, J. D., Richardson, D. L., Kerlavage, A. R., Graham, D. E., Kyrpides, N. C., Fleischmann, R. D., Quackenbush, J., Lee, N. H., Sutton, G. G., Gill, S., Kirkness, E. F., Dougherty, B. A., McKenney, K., Adams, M. D., Loftus, B., Peterson, S., Reich, C. I., McNeil, L. K., Badger, J. H., Glodek, A., Zhou, L., Overbeek, R., Gocayne, J. D., Weidman, J. F., McDonald, L., Utterback, T., Cotton, M. D., Spriggs, T., Artiach, P., Kaine, B. P., Sykes, S. M., Sadow, P. W., D'Andrea, K. P., Bowman, C., Fujii, C., Garland, S. A., Mason, T. M., Olsen, G. J., Fraser, C. M., Smith, H. O., Woese, C. R., and Venter, J. C. (1997) The complete genome sequence of the hyperthermophilic, sulphate-reducing archaeon Archaeoglobus fulgidus. *Nature* **390**, 364-370

8. Burggraf, S., Jannasch, H. W., Nicolaus, B., and Stetter, K. O. (1990) Archaeoglobus profundus sp. nov., represents a new species within the sulfate-reducing archaebacteria. *Systematic and Applied Microbiology* **13**, 5

9. Stokke, R., Hocking, W. P., Steinsbu, B. O., and Steen, I. H. (2013) Complete Genome Sequence of the Thermophilic and Facultatively Chemolithoautotrophic Sulfate Reducer Archaeoglobus sulfaticallidus Strain PM70-1T. *Genome Announc* **1**

10. Huber, H., Jannasch, H., Rachel, R., Fuchs, T., and Stetter, K. O. (1997) Archaeoglobus veneficus sp. nov., a Novel Facultative Chemolithoautotrophic Hyperthermophilic Sulfite Reducer, Isolated from Abyssal Black Smokers. *Systematic and Applied Microbiology* **20**, 7

11. Zaremba-Niedzwiedzka, K., Caceres, E. F., Saw, J. H., Backstrom, D., Juzokaite, L., Vancaester, E., Seitz, K. W., Anantharaman, K., Starnawski, P., Kjeldsen, K. U., Stott, M. B., Nunoura, T., Banfield, J. F., Schramm, A., Baker, B. J., Spang, A., and Ettema, T. J. (2017) Asgard archaea illuminate the origin of eukaryotic cellular complexity. *Nature* **541**, 353-358

12. Evans, P. N., Parks, D. H., Chadwick, G. L., Robbins, S. J., Orphan, V. J., Golding, S. D., and Tyson, G. W. (2015) Methane metabolism in the archaeal phylum Bathyarchaeota revealed by genome-centric metagenomics. *Science* **350**, 434-438

13. Itoh, T., Suzuki, K., Sanchez, P. C., and Nakase, T. (2003) Caldisphaera lagunensis gen. nov., sp. nov., a novel thermoacidophilic crenarchaeote isolated from a hot spring at Mt Maquiling, Philippines. *Int J Syst Evol Microbiol* **53**, 1149-1154

14. Itoh, T., Suzuki, K., Sanchez, P. C., and Nakase, T. (1999) Caldivirga maquilingensis gen. nov., sp. nov., a new genus of rod-shaped crenarchaeote isolated from a hot spring in the Philippines. *Int J Syst Bacteriol* **49 Pt 3**, 1157-1163

15. Mwirichia, R., Alam, I., Rashid, M., Vinu, M., Ba-Alawi, W., Anthony Kamau, A., Kamanda Ngugi, D., Goker, M., Klenk, H. P., Bajic, V., and Stingl, U. (2016) Metabolic traits of an uncultured archaeal lineage--MSBL1--from brine pools of the Red Sea. *Sci Rep* **6**, 19181

16. Giaveno, M. A., Urbieta, M. S., Ulloa, J. R., Toril, E. G., and Donati, E. R. (2013) Physiologic versatility and growth flexibility as the main characteristics of a novel thermoacidophilic Acidianus strain isolated from Copahue geothermal area in Argentina. *Microb Ecol* **65**, 336-346

17. Takami, H., Arai, W., Takemoto, K., Uchiyama, I., and Taniguchi, T. (2015) Functional Classification of Uncultured "Candidatus Caldiarchaeum subterraneum" Using the Maple System. *PLoS One* **10**, e0132994

18. Ugalde, J. A., Narasingarao, P., Kuo, S., Podell, S., and Allen, E. E. (2013) Draft Genome Sequence of "Candidatus Halobonum tyrrellensis" Strain G22, Isolated from the Hypersaline Waters of Lake Tyrrell, Australia. *Genome Announc* **1**

19. Ghai, R., Pasic, L., Fernandez, A. B., Martin-Cuadrado, A. B., Mizuno, C. M., McMahon, K. D., Papke, R. T., Stepanauskas, R., Rodriguez-Brito, B., Rohwer, F., Sanchez-Porro, C., Ventosa, A., and Rodriguez-Valera, F. (2011) New abundant microbial groups in aquatic hypersaline environments. *Sci Rep* **1**, 135

20. Elkins, J. G., Podar, M., Graham, D. E., Makarova, K. S., Wolf, Y., Randau, L., Hedlund, B. P., Brochier-Armanet, C., Kunin, V., Anderson, I., Lapidus, A., Goltsman, E., Barry, K., Koonin, E. V., Hugenholtz, P., Kyrpides, N., Wanner, G., Richardson, P., Keller, M., and Stetter, K. O. (2008) A korarchaeal genome reveals insights into the evolution of the Archaea. *Proc Natl Acad Sci U S A* **105**, 8102-8107

21. Borrel, G., Harris, H. M., Parisot, N., Gaci, N., Tottey, W., Mihajlovski, A., Deane, J., Gribaldo, S., Bardot, O., Peyretaillade, E., Peyret, P., O'Toole, P. W., and Brugere, J. F. (2013) Genome Sequence of "Candidatus Methanomassiliicoccus intestinalis" Issoire-Mx1, a Third Thermoplasmatales-Related Methanogenic Archaeon from Human Feces. *Genome Announc* **1**

22. Borrel, G., Harris, H. M., Tottey, W., Mihajlovski, A., Parisot, N., Peyretaillade, E., Peyret, P., Gribaldo, S., O'Toole, P. W., and Brugere, J. F. (2012) Genome sequence of "Candidatus Methanomethylophilus alvus" Mx1201, a methanogenic archaeon from the human gut belonging to a seventh order of methanogens. *J Bacteriol* **194**, 6944-6945

23. Noel, S. J., Hojberg, O., Urich, T., and Poulsen, M. (2016) Draft Genome Sequence of "Candidatus Methanomethylophilus" sp. 1R26, Enriched from Bovine Rumen, a Methanogenic Archaeon Belonging to the Methanomassiliicoccales Order. *Genome Announc* **4**

24. Haroon, M. F., Hu, S., Shi, Y., Imelfort, M., Keller, J., Hugenholtz, P., Yuan, Z., and Tyson, G. W. (2013) Anaerobic oxidation of methane coupled to nitrate reduction in a novel archaeal lineage. *Nature* **500**, 567-570

25. Berger, S., Frank, J., Dalcin Martins, P., Jetten, M. S. M., and Welte, C. U. (2017) High-Quality Draft Genome Sequence of "Candidatus Methanoperedens sp." Strain BLZ2, a Nitrate-Reducing Anaerobic Methane-Oxidizing Archaeon Enriched in an Anoxic Bioreactor. *Genome Announc* **5**

26. Lang, K., Schuldes, J., Klingl, A., Poehlein, A., Daniel, R., and Brunea, A. (2015) New mode of energy metabolism in the seventh order of methanogens as revealed by comparative genome analysis of "Candidatus methanoplasma termitum". *Appl Environ Microbiol* **81**, 1338-1352

27. Kim, B. K., Jung, M. Y., Yu, D. S., Park, S. J., Oh, T. K., Rhee, S. K., and Kim, J. F. (2011) Genome sequence of an ammonia-oxidizing soil archaeon, "Candidatus Nitrosoarchaeum koreensis" MY1. *J Bacteriol* **193**, 5539-5540

28. Mosier, A. C., Allen, E. E., Kim, M., Ferriera, S., and Francis, C. A. (2012) Genome sequence of "Candidatus Nitrosoarchaeum limnia" BG20, a low-salinity ammonia-oxidizing archaeon from the San Francisco Bay estuary. *J Bacteriol* **194**, 2119-2120

29. Santoro, A. E., Dupont, C. L., Richter, R. A., Craig, M. T., Carini, P., McIlvin, M. R., Yang, Y., Orsi, W. D., Moran, D. M., and Saito, M. A. (2015) Genomic and proteomic characterization of "Candidatus Nitrosopelagicus brevis": an ammonia-oxidizing archaeon from the open ocean. *Proc Natl Acad Sci U S A* **112**, 1173-1178

30. Park, S. J., Kim, J. G., Jung, M. Y., Kim, S. J., Cha, I. T., Kwon, K., Lee, J. H., and Rhee, S. K. (2012) Draft genome sequence of an ammonia-oxidizing archaeon, "Candidatus Nitrosopumilus koreensis" AR1, from marine sediment. *J Bacteriol* **194**, 6940-6941

31. Mosier, A. C., Allen, E. E., Kim, M., Ferriera, S., and Francis, C. A. (2012) Genome sequence of "Candidatus Nitrosopumilus salaria" BD31, an ammonia-oxidizing archaeon from the San Francisco Bay estuary. *J Bacteriol* **194**, 2121-2122

32. Park, S. J., Kim, J. G., Jung, M. Y., Kim, S. J., Cha, I. T., Ghai, R., Martin-Cuadrado, A. B., Rodriguez-Valera, F., and Rhee, S. K. (2012) Draft genome sequence of an ammonia-oxidizing archaeon, "Candidatus Nitrosopumilus sediminis" AR2, from Svalbard in the Arctic Circle. *J Bacteriol* **194**, 6948-6949

33. Spang, A., Poehlein, A., Offre, P., Zumbragel, S., Haider, S., Rychlik, N., Nowka, B., Schmeisser, C., Lebedeva, E. V., Rattei, T., Bohm, C., Schmid, M., Galushko, A., Hatzenpichler, R., Weinmaier, T., Daniel, R., Schleper, C., Spieck, E., Streit, W., and Wagner, M. (2012) The genome of the ammonia-oxidizing Candidatus Nitrososphaera gargensis: insights into metabolic versatility and environmental adaptations. *Environ Microbiol* **14**, 3122-3145

34. Lehtovirta-Morley, L. E., Sayavedra-Soto, L. A., Gallois, N., Schouten, S., Stein, L. Y., Prosser, J. I., and Nicol, G. W. (2016) Identifying Potential Mechanisms Enabling Acidophily in the Ammonia-Oxidizing Archaeon "Candidatus Nitrosotalea devanaterra". *Appl Environ Microbiol* **82**, 2608-2619

35. Laso-Perez, R., Wegener, G., Knittel, K., Widdel, F., Harding, K. J., Krukenberg, V., Meier, D. V., Richter, M., Tegetmeyer, H. E., Riedel, D., Richnow, H. H., Adrian, L., Reemtsma, T., Lechtenfeld, O. J., and Musat, F. (2016) Thermophilic archaea activate butane via alkyl-coenzyme M formation. *Nature* **539**, 396-401

36. Hallam, S. J., Konstantinidis, K. T., Putnam, N., Schleper, C., Watanabe, Y., Sugahara, J., Preston, C., de la Torre, J., Richardson, P. M., and DeLong, E. F. (2006) Genomic analysis of the uncultivated marine crenarchaeote Cenarchaeum symbiosum. *Proc Natl Acad Sci U S A* **103**, 18296-18301

37. Whitman, W. B. (2015) *Bergey's Manual of Systematics of Archaea and Bacteria*

38. Ravin, N. V., Mardanov, A. V., Beletsky, A. V., Kublanov, I. V., Kolganova, T. V., Lebedinsky, A. V., Chernyh, N. A., Bonch-Osmolovskaya, E. A., and Skryabin, K. G. (2009) Complete genome sequence of the anaerobic, protein-degrading hyperthermophilic crenarchaeon Desulfurococcus kamchatkensis. *J Bacteriol* **191**, 2371-2379

39. Anderson, I., Risso, C., Holmes, D., Lucas, S., Copeland, A., Lapidus, A., Cheng, J. F., Bruce, D., Goodwin, L., Pitluck, S., Saunders, E., Brettin, T., Detter, J. C., Han, C., Tapia, R., Larimer, F., Land, M., Hauser, L., Woyke, T., Lovley, D., Kyrpides, N., and Ivanova, N. (2011) Complete genome sequence of Ferroglobus placidus AEDII12DO. *Stand Genomic Sci* **5**, 50-60

40. Golyshina, O. V., Tran, H., Reva, O. N., Lemak, S., Yakunin, A. F., Goesmann, A., Nechitaylo, T. Y., LaCono, V., Smedile, F., Slesarev, A., Rojo, D., Barbas, C., Ferrer, M., Yakimov, M. M., and Golyshin, P. N. (2017) Metabolic and evolutionary patterns in the extremely acidophilic archaeon Ferroplasma acidiphilum Y(T). *Sci Rep* **7**, 3682

41. Perevalova, A. A., Bidzhieva, S., Kublanov, I. V., Hinrichs, K. U., Liu, X. L., Mardanov, A. V., Lebedinsky, A. V., and Bonch-Osmolovskaya, E. A. (2010) Fervidicoccus fontis gen. nov., sp. nov., an anaerobic, thermophilic crenarchaeote from terrestrial hot springs, and proposal of Fervidicoccaceae fam. nov. and Fervidicoccales ord. nov. *Int J Syst Evol Microbiol* **60**, 2082-2088

42. Kashefi, K., Tor, J. M., Holmes, D. E., Gaw Van Praagh, C. V., Reysenbach, A. L., and Lovley, D. R. (2002) Geoglobus ahangari gen. nov., sp. nov., a novel hyperthermophilic archaeon capable of oxidizing organic acids and growing autotrophically on hydrogen with Fe(III) serving as the sole electron acceptor. *Int J Syst Evol Microbiol* **52**, 719-728

43. Baker, B. J., Saw, J. H., Lind, A. E., Lazar, C. S., Hinrichs, K. U., Teske, A. P., and Ettema, T. J. (2016) Genomic inference of the metabolism of cosmopolitan subsurface Archaea, Hadesarchaea. *Nat Microbiol* **1**, 16002

44. Cui, H. L., Sun, F. F., Gao, X., Dong, Y., Xu, X. W., Zhou, Y. G., Liu, H. C., Oren, A., and Zhou, P. J. (2010) Haladaptatus litoreus sp. nov., an extremely halophilic archaeon from a marine solar saltern, and emended description of the genus Haladaptatus. *Int J Syst Evol Microbiol* **60**, 1085-1089

45. Sen, U., Mukherjee, T., Bose, S., Roy, C., Rameez, M. J., Ghosh, W., and Mukhopadhyay, S. K. (2016) Genome Sequence of the Arsenic-Resistant Haladaptatus sp. Strain R4 Isolated from Ramnagar, West Bengal, India. *Genome Announc* **4**

46. Roh, S. W., Nam, Y. D., Chang, H. W., Sung, Y., Kim, K. H., Oh, H. M., and Bae, J. W. (2007) Halalkalicoccus jeotgali sp. nov., a halophilic archaeon from shrimp jeotgal, a traditional Korean fermented seafood. *Int J Syst Evol Microbiol* **57**, 2296-2298

47. Sorokin, D. Y., Kublanov, I. V., Yakimov, M. M., Rijpstra, W. I., and Sinninghe Damste, J. S. (2016) Halanaeroarchaeum sulfurireducens gen. nov., sp. nov., the first obligately anaerobic sulfur-respiring haloarchaeon, isolated from a hypersaline lake. *Int J Syst Evol Microbiol* **66**, 2377-2381

48. Minegishi, H., Echigo, A., Nagaoka, S., Kamekura, M., and Usami, R. (2010) Halarchaeum acidiphilum gen. nov., sp. nov., a moderately acidophilic haloarchaeon isolated from commercial solar salt. *Int J Syst Evol Microbiol* **60**, 2513-2516

49. Makhdoumi-Kakhki, A., Amoozegar, M. A., Bagheri, M., Ramezani, M., and Ventosa, A. (2012) Haloarchaeobius iranensis gen. nov., sp. nov., an extremely halophilic archaeon isolated from a saline lake. *Int J Syst Evol Microbiol* **62**, 1021-1026

50. Sorokin, D. Y., Messina, E., Smedile, F., Roman, P., Damste, J. S. S., Ciordia, S., Mena, M. C., Ferrer, M., Golyshin, P. N., Kublanov, I. V., Samarov, N. I., Toshchakov, S. V., La Cono, V., and Yakimov, M. M. (2017) Discovery of anaerobic lithoheterotrophic haloarchaea, ubiquitous in hypersaline habitats. *ISME J* **11**, 1245-1260

51. Baliga, N. S., Bonneau, R., Facciotti, M. T., Pan, M., Glusman, G., Deutsch, E. W., Shannon, P., Chiu, Y., Weng, R. S., Gan, R. R., Hung, P., Date, S. V., Marcotte, E., Hood, L., and Ng, W. V. (2004) Genome sequence of Haloarcula marismortui: a halophilic archaeon from the Dead Sea. *Genome Res* **14**, 2221-2234

52. Jaakkola, S. T., Pfeiffer, F., Ravantti, J. J., Guo, Q., Liu, Y., Chen, X., Ma, H., Yang, C., Oksanen, H. M., and Bamford, D. H. (2016) The complete genome of a viable archaeum isolated from 123-million-year-old rock salt. *Environ Microbiol* **18**, 565-579

53. Yang, Y., Cui, H. L., Zhou, P. J., and Liu, S. J. (2006) Halobacterium jilantaiense sp. nov., a halophilic archaeon isolated from a saline lake in Inner Mongolia, China. *Int J Syst Evol Microbiol* **56**, 2353-2355

54. DasSarma, S., Berquist, B. R., Coker, J. A., DasSarma, P., and Muller, J. A. (2006) Post-genomics of the model haloarchaeon Halobacterium sp. NRC-1. *Saline Systems* **2**, 3

55. Oren, A., Gurevich, P., Gemmell, R. T., and Teske, A. (1995) Halobaculum gomorrense gen. nov., sp. nov., a novel extremely halophilic archaeon from the Dead Sea. *Int J Syst Bacteriol* **45**, 747-754

56. Cui, H. L., Yang, X., Gao, X., and Xu, X. W. (2011) Halobellus clavatus gen. nov., sp. nov. and Halorientalis regularis gen. nov., sp. nov., two new members of the family Halobacteriaceae. *Int J Syst Evol Microbiol* **61**, 2682-2689

57. Xu, X. W., Wu, M., Zhou, P. J., and Liu, S. J. (2005) Halobiforma lacisalsi sp. nov., isolated from a salt lake in China. *Int J Syst Evol Microbiol* **55**, 1949-1952

58. Hezayen, F. F., Tindall, B. J., Steinbuchel, A., and Rehm, B. H. (2002) Characterization of a novel halophilic archaeon, Halobiforma haloterrestris gen. nov., sp. nov., and transfer of Natronobacterium nitratireducens to Halobiforma nitratireducens comb. nov. *Int J Syst Evol Microbiol* **52**, 2271-2280

59. Goh, F., Leuko, S., Allen, M. A., Bowman, J. P., Kamekura, M., Neilan, B. A., and Burns, B. P. (2006) Halococcus hamelinensis sp. nov., a novel halophilic archaeon isolated from stromatolites in Shark Bay, Australia. *Int J Syst Evol Microbiol* **56**, 1323-1329

60. Steber, J., and Schleifer, K. H. (1975) Halococcus morrhuae: a sulfated heteropolysaccharide as the structural component of the bacterial cell wall. *Arch Microbiol* **105**, 173-177

61. Montero, C. G., Ventosa, A., Rodriguez-Valera, F., Kates, M., Moldoveanu, N., and Ruiz-Berraquero, F. (1989) Halococcus saccharolyticus sp. nov., a New Species of Extremely Halophilic Non-alkaliphilic Cocci. *Systematic and Applied Microbiology* **12**, 5

62. Xu, X. W., Wu, Y. H., Wang, C. S., Oren, A., Zhou, P. J., and Wu, M. (2007) Haloferax larsenii sp. nov., an extremely halophilic archaeon from a solar saltern. *Int J Syst Evol Microbiol* **57**, 717-720

63. Han, J., Zhang, F., Hou, J., Liu, X., Li, M., Liu, H., Cai, L., Zhang, B., Chen, Y., Zhou, J., Hu, S., and Xiang, H. (2012) Complete genome sequence of the metabolically versatile halophilic archaeon Haloferax mediterranei, a poly(3-hydroxybutyrate-co-3-hydroxyvalerate) producer. *J Bacteriol* **194**, 4463-4464

64. Hartman, A. L., Norais, C., Badger, J. H., Delmas, S., Haldenby, S., Madupu, R., Robinson, J., Khouri, H., Ren, Q., Lowe, T. M., Maupin-Furlow, J., Pohlschroder, M., Daniels, C., Pfeiffer, F., Allers, T., and Eisen, J. A. (2010) The complete genome sequence of Haloferax volcanii DS2, a model archaeon. *PLoS One* **5**, e9605

65. Montalvo-Rodriguez, R., Vreeland, R. H., Oren, A., Kessel, M., Betancourt, C., and Lopez-Garriga, J. (1998) Halogeometricum borinquense gen. nov., sp. nov., a novel halophilic archaeon from Puerto Rico. *Int J Syst Bacteriol* **48 Pt 4**, 1305-1312

66. Cui, H. L., Yang, X., Gao, X., Li, X. Y., Xu, X. W., Zhou, Y. G., Liu, H. C., and Zhou, P. J. (2010) Halogeometricum rufum sp. nov., a halophilic archaeon from a marine solar saltern, and emended description of the genus Halogeometricum. *Int J Syst Evol Microbiol* **60**, 2613-2617

67. Cui, H. L., Yang, X., Gao, X., and Xu, X. W. (2011) Halogranum gelatinilyticum sp. nov. and Halogranum amylolyticum sp. nov., isolated from a marine solar saltern, and emended description of the genus Halogranum. *Int J Syst Evol Microbiol* **61**, 911-915

68. Kim, K. K., Lee, K. C., and Lee, J. S. (2011) Halogranum salarium sp. nov., a halophilic archaeon isolated from sea salt. *Syst Appl Microbiol* **34**, 576-580

69. Mou, Y. Z., Qiu, X. X., Zhao, M. L., Cui, H. L., Oh, D., and Dyall-Smith, M. L. (2012) Halohasta litorea gen. nov. sp. nov., and Halohasta litchfieldiae sp. nov., isolated from the Daliang aquaculture farm, China and from Deep Lake, Antarctica, respectively. *Extremophiles* **16**, 895-901

70. Cui, H. L., Gao, X., Yang, X., and Xu, X. W. (2011) Halolamina pelagica gen. nov., sp. nov., a new member of the family Halobacteriaceae. *Int J Syst Evol Microbiol* **61**, 1617-1621

71. Oren, A., Elevi, R., Watanabe, S., Ihara, K., and Corcelli, A. (2002) Halomicrobium mukohataei gen. nov., comb. nov., and emended description of Halomicrobium mukohataei. *Int J Syst Evol Microbiol* **52**, 1831-1835

72. Yang, X., and Cui, H. L. (2012) Halomicrobium zhouii sp. nov., a halophilic archaeon from a marine solar saltern. *Int J Syst Evol Microbiol* **62**, 1235-1240

73. Cui, H. L., Li, X. Y., Gao, X., Xu, X. W., Zhou, Y. G., Liu, H. C., Oren, A., and Zhou, P. J. (2010) Halopelagius inordinatus gen. nov., sp. nov., a new member of the family Halobacteriaceae isolated from a marine solar saltern. *Int J Syst Evol Microbiol* **60**, 2089-2093

74. Zhang, X., Zhang, W. Y., Shen, A. H., Huo, Y. Y., Zhu, X. F., and Wu, M. (2013) Halopelagius longus sp. nov., a member of the family Halobacteriaceae isolated from a salt mine, and emended description of the genus Halopelagius. *Int J Syst Evol Microbiol* **63**, 3585-3590

75. Amoozegar, M. A., Makhdoumi-Kakhki, A., Mehrshad, M., Fazeli, S. A., and Ventosa, A. (2013) Halopenitus malekzadehii sp. nov., an extremely halophilic archaeon isolated from a salt lake. *Int J Syst Evol Microbiol* **63**, 3232-3236

76. Amoozegar, M. A., Makhdoumi-Kakhki, A., Shahzadeh Fazeli, S. A., Azarbaijani, R., and Ventosa, A. (2012) Halopenitus persicus gen. nov., sp. nov., an archaeon from an inland salt lake. *Int J Syst Evol Microbiol* **62**, 1932-1936

77. Zhang, W. Y., Meng, Y., Zhu, X. F., and Wu, M. (2013) Halopiger salifodinae sp. nov., an extremely halophilic archaeon isolated from a salt mine. *Int J Syst Evol Microbiol* **63**, 3563-3567

78. Gutierrez, M. C., Castillo, A. M., Kamekura, M., Xue, Y., Ma, Y., Cowan, D. A., Jones, B. E., Grant, W. D., and Ventosa, A. (2007) Halopiger xanaduensis gen. nov., sp. nov., an extremely halophilic archaeon isolated from saline Lake Shangmatala in Inner Mongolia, China. *Int J Syst Evol Microbiol* **57**, 1402-1407

79. Bolhuis, H., Palm, P., Wende, A., Falb, M., Rampp, M., Rodriguez-Valera, F., Pfeiffer, F., and Oesterhelt, D. (2006) The genome of the square archaeon Haloquadratum walsbyi : life at the limits of water activity. *BMC Genomics* **7**, 169

80. Tully, B. J., Emerson, J. B., Andrade, K., Brocks, J. J., Allen, E. E., Banfield, J. F., and Heidelberg, K. B. (2015) De novo sequences of Haloquadratum walsbyi from Lake Tyrrell, Australia, reveal a variable genomic landscape. *Archaea* **2015**, 875784

81. Antunes, A., Taborda, M., Huber, R., Moissl, C., Nobre, M. F., and da Costa, M. S. (2008) Halorhabdus tiamatea sp. nov., a non-pigmented, extremely halophilic archaeon from a deep-sea, hypersaline anoxic basin of the Red Sea, and emended description of the genus Halorhabdus. *Int J Syst Evol Microbiol* **58**, 215-220

82. Cui, H. L., Tohty, D., Zhou, P. J., and Liu, S. J. (2006) Halorubrum lipolyticum sp. nov. and Halorubrum aidingense sp. nov., isolated from two salt lakes in Xin-Jiang, China. *Int J Syst Evol Microbiol* **56**, 1631-1634

83. Corral, P., de la Haba, R. R., Sanchez-Porro, C., Ali Amoozegar, M., Thane Papke, R., and Ventosa, A. (2016) Halorubrum halodurans sp. nov., an extremely halophilic archaeon isolated from a hypersaline lake. *Int J Syst Evol Microbiol* **66**, 435-444

84. Anderson, I. J., DasSarma, P., Lucas, S., Copeland, A., Lapidus, A., Del Rio, T. G., Tice, H., Dalin, E., Bruce, D. C., Goodwin, L., Pitluck, S., Sims, D., Brettin, T. S., Detter, J. C., Han, C. S., Larimer, F., Hauser, L., Land, M., Ivanova, N., Richardson, P., Cavicchioli, R., DasSarma, S., Woese, C. R., and Kyrpides, N. C. (2016) Complete genome sequence of the Antarctic Halorubrum lacusprofundi type strain ACAM 34. *Stand Genomic Sci* **11**, 70

85. Corral, P., de la Haba, R. R., Sanchez-Porro, C., Amoozegar, M. A., Papke, R. T., and Ventosa, A. (2015) Halorubrum persicum sp. nov., an extremely halophilic archaeon isolated from sediment of a hypersaline lake. *Int J Syst Evol Microbiol* **65**, 1770-1778

86. Burguener, G. F., Maldonado, M. J., Revale, S., Fernandez Do Porto, D., Rascovan, N., Vazquez, M., Farias, M. E., Marti, M. A., and Turjanski, A. G. (2014) Draft Genome Sequence of the Polyextremophilic Halorubrum sp. Strain AJ67, Isolated from Hyperarsenic Lakes in the Argentinian Puna. *Genome Announc* **2**

87. Savage, K. N., Krumholz, L. R., Oren, A., and Elshahed, M. S. (2008) Halosarcina pallida gen. nov., sp. nov., a halophilic archaeon from a low-salt, sulfide-rich spring. *Int J Syst Evol Microbiol* **58**, 856-860

88. Vreeland, R. H., Straight, S., Krammes, J., Dougherty, K., Rosenzweig, W. D., and Kamekura, M. (2002) Halosimplex carlsbadense gen. nov., sp. nov., a unique halophilic archaeon, with three 16S rRNA genes, that grows only in defined medium with glycerol and acetate or pyruvate. *Extremophiles* **6**, 445-452

89. Castillo, A. M., Gutierrez, M. C., Kamekura, M., Xue, Y., Ma, Y., Cowan, D. A., Jones, B. E., Grant, W. D., and Ventosa, A. (2006) Halostagnicola larsenii gen. nov., sp. nov., an extremely halophilic archaeon from a saline lake in Inner Mongolia, China. *Int J Syst Evol Microbiol* **56**, 1519-1524

90. Kanekar, S. P., Saxena, N., Pore, S. D., Arora, P., Kanekar, P. P., and Dhakephalkar, P. K. (2015) Draft Genome Sequence of Halostagnicola sp. A56, an Extremely Halophilic Archaeon Isolated from the Andaman Islands. *Genome Announc* **3**

91. Wang, S., Yang, Q., Liu, Z. H., Sun, L., Wei, D., Zhang, J. Z., Song, J. Z., and Yuan, H. F. (2010) Haloterrigena daqingensis sp. nov., an extremely haloalkaliphilic archaeon isolated from a saline-alkaline soil. *Int J Syst Evol Microbiol* **60**, 2267-2271

92. Cui, H. L., Tohty, D., Zhou, P. J., and Liu, S. J. (2006) Haloterrigena longa sp. nov. and Haloterrigena limicola sp. nov., extremely halophilic archaea isolated from a salt lake. *Int J Syst Evol Microbiol* **56**, 1837-1840

93. Saunders, E., Tindall, B. J., Fahnrich, R., Lapidus, A., Copeland, A., Del Rio, T. G., Lucas, S., Chen, F., Tice, H., Cheng, J. F., Han, C., Detter, J. C., Bruce, D., Goodwin, L., Chain, P., Pitluck, S., Pati, A., Ivanova, N., Mavromatis, K., Chen, A., Palaniappan, K., Land, M., Hauser, L., Chang, Y. J., Jeffries, C. D., Brettin, T., Rohde, M., Goker, M., Bristow, J., Eisen, J. A., Markowitz, V., Hugenholtz, P., Klenk, H. P., and Kyrpides, N. C. (2010) Complete genome sequence of Haloterrigena turkmenica type strain (4k). *Stand Genomic Sci* **2**, 107-116

94. Makhdoumi-Kakhki, A., Amoozegar, M. A., and Ventosa, A. (2012) Halovenus aranensis gen. nov., sp. nov., an extremely halophilic archaeon from Aran-Bidgol salt lake. *Int J Syst Evol Microbiol* **62**, 1331-1336

95. Castillo, A. M., Gutierrez, M. C., Kamekura, M., Xue, Y., Ma, Y., Cowan, D. A., Jones, B. E., Grant, W. D., and Ventosa, A. (2007) Halovivax ruber sp. nov., an extremely halophilic archaeon isolated from Lake Xilinhot, Inner Mongolia, China. *Int J Syst Evol Microbiol* **57**, 1024-1027

96. Brugger, K., Chen, L., Stark, M., Zibat, A., Redder, P., Ruepp, A., Awayez, M., She, Q., Garrett, R. A., and Klenk, H. P. (2007) The genome of Hyperthermus butylicus: a sulfur-reducing, peptide fermenting, neutrophilic Crenarchaeote growing up to 108 degrees C. *Archaea* **2**, 127-135

97. Paper, W., Jahn, U., Hohn, M. J., Kronner, M., Nather, D. J., Burghardt, T., Rachel, R., Stetter, K. O., and Huber, H. (2007) Ignicoccus hospitalis sp. nov., the host of 'Nanoarchaeum equitans'. *Int J Syst Evol Microbiol* **57**, 803-808

98. Huber, H., Burggraf, S., Mayer, T., Wyschkony, I., Rachel, R., and Stetter, K. O. (2000) Ignicoccus gen. nov., a novel genus of hyperthermophilic, chemolithoautotrophic Archaea, represented by two new species, Ignicoccus islandicus sp nov and Ignicoccus pacificus sp nov. and Ignicoccus pacificus sp. nov. *Int J Syst Evol Microbiol* **50 Pt 6**, 2093-2100

99. Niederberger, T. D., Gotz, D. K., McDonald, I. R., Ronimus, R. S., and Morgan, H. W. (2006) Ignisphaera aggregans gen. nov., sp. nov., a novel hyperthermophilic crenarchaeote isolated from hot springs in Rotorua and Tokaanu, New Zealand. *Int J Syst Evol Microbiol* **56**, 965-971

100. Zhang, C. L., Xie, W., Martin-Cuadrado, A. B., and Rodriguez-Valera, F. (2015) Marine Group II Archaea, potentially important players in the global ocean carbon cycle. *Front Microbiol* **6**, 1108

101. Auernik, K. S., Maezato, Y., Blum, P. H., and Kelly, R. M. (2008) The genome sequence of the metal-mobilizing, extremely thermoacidophilic archaeon Metallosphaera sedula provides insights into bioleaching-associated metabolism. *Appl Environ Microbiol* **74**, 682-692

102. Kozubal, M. A., Dlakic, M., Macur, R. E., and Inskeep, W. P. (2011) Terminal oxidase diversity and function in "Metallosphaera yellowstonensis": gene expression and protein modeling suggest mechanisms of Fe(II) oxidation in the sulfolobales. *Appl Environ Microbiol* **77**, 1844-1853

103. Tejerizo, G. T., Kim, Y. S., Maus, I., Wibberg, D., Winkler, A., Off, S., Puhler, A., Scherer, P., and Schluter, A. (2017) Genome sequence of Methanobacterium congolense strain Buetzberg, a hydrogenotrophic, methanogenic archaeon, isolated from a mesophilic industrial-scale biogas plant utilizing bio-waste. *J Biotechnol* **247**, 1-5

104. Cadillo-Quiroz, H., Brauer, S. L., Goodson, N., Yavitt, J. B., and Zinder, S. H. (2014) Methanobacterium paludis sp. nov. and a novel strain of Methanobacterium lacus isolated from northern peatlands. *Int J Syst Evol Microbiol* **64**, 1473-1480

105. Maus, I., Wibberg, D., Stantscheff, R., Cibis, K., Eikmeyer, F. G., Konig, H., Puhler, A., and Schluter, A. (2013) Complete genome sequence of the hydrogenotrophic Archaeon Methanobacterium sp. Mb1 isolated from a production-scale biogas plant. *J Biotechnol* **168**, 734-736

106. Poehlein, A., and Seedorf, H. (2016) Draft Genome Sequences of Methanobrevibacter curvatus DSM11111, Methanobrevibacter cuticularis DSM11139, Methanobrevibacter filiformis DSM11501, and Methanobrevibacter oralis DSM7256. *Genome Announc* **4**

107. Kelly, W. J., Pacheco, D. M., Li, D., Attwood, G. T., Altermann, E., and Leahy, S. C. (2016) The complete genome sequence of the rumen methanogen Methanobrevibacter millerae SM9. *Stand Genomic Sci* **11**, 49

108. Leahy, S. C., Kelly, W. J., Altermann, E., Ronimus, R. S., Yeoman, C. J., Pacheco, D. M., Li, D., Kong, Z., McTavish, S., Sang, C., Lambie, S. C., Janssen, P. H., Dey, D., and Attwood, G. T. (2010) The genome sequence of the rumen methanogen Methanobrevibacter ruminantium reveals new possibilities for controlling ruminant methane emissions. *PLoS One* **5**, e8926

109. Samuel, B. S., Hansen, E. E., Manchester, J. K., Coutinho, P. M., Henrissat, B., Fulton, R., Latreille, P., Kim, K., Wilson, R. K., and Gordon, J. I. (2007) Genomic and metabolic adaptations of Methanobrevibacter smithii to the human gut. *Proc Natl Acad Sci U S A* **104**, 10643-10648

110. Jeanthon, C., L'Haridon, S., Reysenbach, A. L., Vernet, M., Messner, P., Sleytr, U. B., and Prieur, D. (1998) Methanococcus infernus sp. nov., a novel hyperthermophilic lithotrophic methanogen isolated from a deep-sea hydrothermal vent. *Int J Syst Bacteriol* **48 Pt 3**, 913-919

111. Bult, C. J., White, O., Olsen, G. J., Zhou, L., Fleischmann, R. D., Sutton, G. G., Blake, J. A., FitzGerald, L. M., Clayton, R. A., Gocayne, J. D., Kerlavage, A. R., Dougherty, B. A., Tomb, J. F., Adams, M. D., Reich, C. I., Overbeek, R., Kirkness, E. F., Weinstock, K. G., Merrick, J. M., Glodek, A., Scott, J. L., Geoghagen, N. S., and Venter, J. C. (1996) Complete genome sequence of the methanogenic archaeon, Methanococcus jannaschii. *Science* **273**, 1058-1073

112. Sakai, S., Conrad, R., Liesack, W., and Imachi, H. (2010) Methanocella arvoryzae sp. nov., a hydrogenotrophic methanogen isolated from rice field soil. *Int J Syst Evol Microbiol* **60**, 2918-2923

113. Sakai, S., Imachi, H., Hanada, S., Ohashi, A., Harada, H., and Kamagata, Y. (2008) Methanocella paludicola gen. nov., sp. nov., a methane-producing archaeon, the first isolate of the lineage 'Rice Cluster I', and proposal of the new archaeal order Methanocellales ord. nov. *Int J Syst Evol Microbiol* **58**, 929-936

114. Franzmann, P. D., Springer, N., Ludwig, W., Conway De Macario, E., and Rohde, M. (1992) A Methanogenic Archaeon from Ace Lake, Antarctica: Methanococcoides burtonii sp. nov. *Systematic and Applied Microbiology* **15**, 9

115. Kendall, M. M., Liu, Y., Sieprawska-Lupa, M., Stetter, K. O., Whitman, W. B., and Boone, D. R. (2006) Methanococcus aeolicus sp. nov., a mesophilic, methanogenic archaeon from shallow and deep marine sediments. *Int J Syst Evol Microbiol* **56**, 1525-1529

116. Sarmiento, F., Mrazek, J., and Whitman, W. B. (2013) Genome-scale analysis of gene function in the hydrogenotrophic methanogenic archaeon Methanococcus maripaludis. *Proc Natl Acad Sci U S A* **110**, 4726-4731

117. Wood, A. G., Whitman, W. B., and Konisky, J. (1989) Isolation and characterization of an archaebacterial viruslike particle from Methanococcus voltae A3. *J Bacteriol* **171**, 93-98

118. Anderson, I. J., Sieprawska-Lupa, M., Goltsman, E., Lapidus, A., Copeland, A., Glavina Del Rio, T., Tice, H., Dalin, E., Barry, K., Pitluck, S., Hauser, L., Land, M., Lucas, S., Richardson, P., Whitman, W. B., and Kyrpides, N. C. (2009) Complete genome sequence of Methanocorpusculum labreanum type strain Z. *Stand Genomic Sci* **1**, 197-203

119. Maus, I., Wibberg, D., Stantscheff, R., Eikmeyer, F. G., Seffner, A., Boelter, J., Szczepanowski, R., Blom, J., Jaenicke, S., Konig, H., Puhler, A., and Schluter, A. (2012) Complete genome sequence of the hydrogenotrophic, methanogenic archaeon Methanoculleus bourgensis strain MS2(T), Isolated from a sewage sludge digester. *J Bacteriol* **194**, 5487-5488

120. Anderson, I. J., Sieprawska-Lupa, M., Lapidus, A., Nolan, M., Copeland, A., Glavina Del Rio, T., Tice, H., Dalin, E., Barry, K., Saunders, E., Han, C., Brettin, T., Detter, J. C., Bruce, D., Mikhailova, N., Pitluck, S., Hauser, L., Land, M., Lucas, S., Richardson, P., Whitman, W. B., and Kyrpides, N. C. (2009) Complete genome sequence of Methanoculleus marisnigri Romesser et al. 1981 type strain JR1. *Stand Genomic Sci* **1**, 189-196

121. Zellner, G., Boone, D. R., Keswani, J., Whitman, W. B., Woese, C. R., Hagelstein, A., Tindall, B. J., and Stackebrandt, E. (1999) Reclassification of Methanogenium tationis and Methanogenium liminatans as Methanofollis tationis gen. nov., comb. nov. and Methanofollis liminatans comb. nov. and description of a new strain of Methanofollis liminatans. *Int J Syst Bacteriol* **49 Pt 1**, 247-255

122. Li, Y., Leahy, S. C., Jeyanathan, J., Henderson, G., Cox, F., Altermann, E., Kelly, W. J., Lambie, S. C., Janssen, P. H., Rakonjac, J., and Attwood, G. T. (2016) The complete genome sequence of the methanogenic archaeon ISO4-H5 provides insights into the methylotrophic lifestyle of a ruminal representative of the Methanomassiliicoccales. *Stand Genomic Sci* **11**, 59

123. Lipus, D., Vikram, A., Ross, D. E., and Bibby, K. (2016) Draft Genome Sequence of Methanohalophilus mahii Strain DAL1 Reconstructed from a Hydraulic Fracturing-Produced Water Metagenome. *Genome Announc* **4**

124. Mochimaru, H., Tamaki, H., Hanada, S., Imachi, H., Nakamura, K., Sakata, S., and Kamagata, Y. (2009) Methanolobus profundi sp. nov., a methylotrophic methanogen isolated from deep subsurface sediments in a natural gas field. *Int J Syst Evol Microbiol* **59**, 714-718

125. Zhang, G., Jiang, N., Liu, X., and Dong, X. (2008) Methanogenesis from methanol at low temperatures by a novel psychrophilic methanogen, "Methanolobus psychrophilus" sp. nov., prevalent in Zoige wetland of the Tibetan plateau. *Appl Environ Microbiol* **74**, 6114-6120

126. König, H., and Stetter, K. O. (1982) Isolation and characterization of Methanolobus tindarius, sp. nov., a coccoid methanogen growing only on methanol and methylamines. *ZbI. Bakt, Hyg., I. Abt, Orig. C* **3**, 13

127. Lomans, B. P., Maas, R., Luderer, R., Op den Camp, H. J., Pol, A., van der Drift, C., and Vogels, G. D. (1999) Isolation and characterization of Methanomethylovorans hollandica gen. nov., sp. nov., isolated from freshwater sediment, a methylotrophic methanogen able to grow on dimethyl sulfide and methanethiol. *Appl Environ Microbiol* **65**, 3641-3650

128. Goker, M., Lu, M., Fiebig, A., Nolan, M., Lapidus, A., Tice, H., Del Rio, T. G., Cheng, J. F., Han, C., Tapia, R., Goodwin, L. A., Pitluck, S., Liolios, K., Mavromatis, K., Pagani, I., Ivanova, N., Mikhailova, N., Pati, A., Chen, A., Palaniappan, K., Land, M., Mayilraj, S., Rohde, M., Detter, J. C., Bunk, B., Spring, S., Wirth, R., Woyke, T., Bristow, J., Eisen, J. A., Markowitz, V., Hugenholtz, P., Kyrpides, N. C., and Klenk, H. P. (2014) Genome sequence of the mud-dwelling archaeon Methanoplanus limicola type strain (DSM 2279(T)), reclassification of Methanoplanus petrolearius as Methanolacinia petrolearia and emended descriptions of the genera Methanoplanus and Methanolacinia. *Stand Genomic Sci* **9**, 1076-1088

129. Ollivier, B., Cayol, J. L., Patel, B. K., Magot, M., Fardeau, M. L., and Garcia, J. L. (1997) Methanoplanus petrolearius sp. nov., a novel methanogenic bacterium from an oil-producing well. *FEMS Microbiol Lett* **147**, 51-56

130. Slesarev, A. I., Mezhevaya, K. V., Makarova, K. S., Polushin, N. N., Shcherbinina, O. V., Shakhova, V. V., Belova, G. I., Aravind, L., Natale, D. A., Rogozin, I. B., Tatusov, R. L., Wolf, Y. I., Stetter, K. O., Malykh, A. G., Koonin, E. V., and Kozyavkin, S. A. (2002) The complete genome of hyperthermophile Methanopyrus kandleri AV19 and monophyly of archaeal methanogens. *Proc Natl Acad Sci U S A* **99**, 4644-4649

131. Brauer, S. L., Cadillo-Quiroz, H., Ward, R. J., Yavitt, J. B., and Zinder, S. H. (2011) Methanoregula boonei gen. nov., sp. nov., an acidiphilic methanogen isolated from an acidic peat bog. *Int J Syst Evol Microbiol* **61**, 45-52

132. Yashiro, Y., Sakai, S., Ehara, M., Miyazaki, M., Yamaguchi, T., and Imachi, H. (2011) Methanoregula formicica sp. nov., a methane-producing archaeon isolated from methanogenic sludge. *Int J Syst Evol Microbiol* **61**, 53-59

133. Barber, R. D., Zhang, L., Harnack, M., Olson, M. V., Kaul, R., Ingram-Smith, C., and Smith, K. S. (2011) Complete genome sequence of Methanosaeta concilii, a specialist in aceticlastic methanogenesis. *J Bacteriol* **193**, 3668-3669

134. Ma, K., Liu, X., and Dong, X. (2006) Methanosaeta harundinacea sp. nov., a novel acetate-scavenging methanogen isolated from a UASB reactor. *Int J Syst Evol Microbiol* **56**, 127-131

135. Kamagata, Y., Kawasaki, H., Oyaizu, H., Nakamura, K., Mikami, E., Endo, G., Koga, Y., and Yamasato, K. (1992) Characterization of three thermophilic strains of Methanothrix ("Methanosaeta") thermophila sp. nov. and rejection of Methanothrix ("Methanosaeta") thermoacetophila. *Int J Syst Bacteriol* **42**, 463-468

136. Sorokin, D. Y., Abbas, B., Merkel, A. Y., Rijpstra, W. I., Damste, J. S., Sukhacheva, M. V., and van Loosdrecht, M. C. (2015) Methanosalsum natronophilum sp. nov., and Methanocalculus alkaliphilus sp. nov., haloalkaliphilic methanogens from hypersaline soda lakes. *Int J Syst Evol Microbiol* **65**, 3739-3745

137. Rother, M., and Metcalf, W. W. (2004) Anaerobic growth of Methanosarcina acetivorans C2A on carbon monoxide: an unusual way of life for a methanogenic archaeon. *Proc Natl Acad Sci U S A* **101**, 16929-16934

138. Maeder, D. L., Anderson, I., Brettin, T. S., Bruce, D. C., Gilna, P., Han, C. S., Lapidus, A., Metcalf, W. W., Saunders, E., Tapia, R., and Sowers, K. R. (2006) The Methanosarcina barkeri genome: comparative analysis with Methanosarcina acetivorans and Methanosarcina mazei reveals extensive rearrangement within methanosarcinal genomes. *J Bacteriol* **188**, 7922-7931

139. Shimizu, S., Upadhye, R., Ishijima, Y., and Naganuma, T. (2011) Methanosarcina horonobensis sp. nov., a methanogenic archaeon isolated from a deep subsurface Miocene formation. *Int J Syst Evol Microbiol* **61**, 2503-2507

140. Simankovaa, M. V., Parshinaa, S. N., Tourovaa, T. P., Kolganovab, T. V., Zehnderc, A. J. B., and Nozhevnikovaa, A. N. (2001) Methanosarcina lacustris sp. nov., a New Psychrotolerant Methanogenic Archaeon from Anoxic Lake Sediments. *Systematic and Applied Microbiology* **24**, 6

141. Jager, D., Sharma, C. M., Thomsen, J., Ehlers, C., Vogel, J., and Schmitz, R. A. (2009) Deep sequencing analysis of the Methanosarcina mazei Go1 transcriptome in response to nitrogen availability. *Proc Natl Acad Sci U S A* **106**, 21878-21882

142. Biavati, B., Vasta, M., and Ferry, J. G. (1988) Isolation and characterization of "Methanosphaera cuniculi" sp. nov. *Appl Environ Microbiol* **54**, 768-771

143. Hoedt, E. C., Cuiv, P. O., Evans, P. N., Smith, W. J., McSweeney, C. S., Denman, S. E., and Morrison, M. (2016) Differences down-under: alcohol-fueled methanogenesis by archaea present in Australian macropodids. *ISME J* **10**, 2376-2388

144. Fricke, W. F., Seedorf, H., Henne, A., Kruer, M., Liesegang, H., Hedderich, R., Gottschalk, G., and Thauer, R. K. (2006) The genome sequence of Methanosphaera stadtmanae reveals why this human intestinal archaeon is restricted to methanol and H2 for methane formation and ATP synthesis. *J Bacteriol* **188**, 642-658

145. Cadillo-Quiroz, H., Browne, P., Kyrpides, N., Woyke, T., Goodwin, L., Detter, C., Yavitt, J. B., and Zinder, S. H. (2015) Complete Genome Sequence of Methanosphaerula palustris E1-9CT, a Hydrogenotrophic Methanogen Isolated from a Minerotrophic Fen Peatland. *Genome Announc* **3**

146. Gunsalus, R. P., Cook, L. E., Crable, B., Rohlin, L., McDonald, E., Mouttaki, H., Sieber, J. R., Poweleit, N., Zhou, H., Lapidus, A. L., Daligault, H. E., Land, M., Gilna, P., Ivanova, N., Kyrpides, N., Culley, D. E., and McInerney, M. J. (2016) Complete genome sequence of Methanospirillum hungatei type strain JF1. *Stand Genomic Sci* **11**, 2

147. de Poorter, L. M., Geerts, W. J., and Keltjens, J. T. (2007) Coupling of Methanothermobacter thermautotrophicus methane formation and growth in fed-batch and continuous cultures under different H2 gassing regimens. *Appl Environ Microbiol* **73**, 740-749

148. Anderson, I., Djao, O. D., Misra, M., Chertkov, O., Nolan, M., Lucas, S., Lapidus, A., Del Rio, T. G., Tice, H., Cheng, J. F., Tapia, R., Han, C., Goodwin, L., Pitluck, S., Liolios, K., Ivanova, N., Mavromatis, K., Mikhailova, N., Pati, A., Brambilla, E., Chen, A., Palaniappan, K., Land, M., Hauser, L., Chang, Y. J., Jeffries, C. D., Sikorski, J., Spring, S., Rohde, M., Eichinger, K., Huber, H., Wirth, R., Goker, M., Detter, J. C., Woyke, T., Bristow, J., Eisen, J. A., Markowitz, V., Hugenholtz, P., Klenk, H. P., and Kyrpides, N. C. (2010) Complete genome sequence of Methanothermus fervidus type strain (V24S). *Stand Genomic Sci* **3**, 315-324

149. Munson-McGee, J. H., Field, E. K., Bateson, M., Rooney, C., Stepanauskas, R., and Young, M. J. (2015) Nanoarchaeota, Their Sulfolobales Host, and Nanoarchaeota Virus Distribution across Yellowstone National Park Hot Springs. *Appl Environ Microbiol* **81**, 7860-7868

150. Waters, E., Hohn, M. J., Ahel, I., Graham, D. E., Adams, M. D., Barnstead, M., Beeson, K. Y., Bibbs, L., Bolanos, R., Keller, M., Kretz, K., Lin, X., Mathur, E., Ni, J., Podar, M., Richardson, T., Sutton, G. G., Simon, M., Soll, D., Stetter, K. O., Short, J. M., and Noordewier, M. (2003) The genome of Nanoarchaeum equitans: insights into early archaeal evolution and derived parasitism. *Proc Natl Acad Sci U S A* **100**, 12984-12988

151. Castillo, A. M., Gutierrez, M. C., Kamekura, M., Xue, Y., Ma, Y., Cowan, D. A., Jones, B. E., Grant, W. D., and Ventosa, A. (2006) Natrinema ejinorense sp. nov., isolated from a saline lake in Inner Mongolia, China. *Int J Syst Evol Microbiol* **56**, 2683-2687

152. McGenity, T. J., Gemmell, R. T., and Grant, W. D. (1998) Proposal of a new halobacterial genus Natrinema gen. nov., with two species Natrinema pellirubrum nom. nov. and Natrinema pallidum nom. nov. *Int J Syst Bacteriol* **48 Pt 4**, 1187-1196

153. Albuquerque, L., Taborda, M., La Cono, V., Yakimov, M., and da Costa, M. S. (2012) Natrinema salaciae sp. nov., a halophilic archaeon isolated from the deep, hypersaline anoxic Lake Medee in the Eastern Mediterranean Sea. *Syst Appl Microbiol* **35**, 368-373

154. Feng, J., Liu, B., Zhang, Z., Ren, Y., Li, Y., Gan, F., Huang, Y., Chen, X., Shen, P., Wang, L., Tang, B., and Tang, X. F. (2012) The complete genome sequence of Natrinema sp. J7-2, a haloarchaeon capable of growth on synthetic media without amino acid supplements. *PLoS One* **7**, e41621

155. Xin, H., Itoh, T., Zhou, P., Suzuki, K., Kamekura, M., and Nakase, T. (2000) Natrinema versiforme sp. nov., an extremely halophilic archaeon from Aibi salt lake, Xinjiang, China. *Int J Syst Evol Microbiol* **50 Pt 3**, 1297-1303

156. Shimane, Y., Nagaoka, S., Minegishi, H., Kamekura, M., Echigo, A., Hatada, Y., Ito, T., and Usami, R. (2013) Natronoarchaeum philippinense sp. nov., a haloarchaeon isolated from commercial solar salt. *Int J Syst Evol Microbiol* **63**, 920-924

157. Ruiz-Romero, E., Sanchez-Lopez, K. B., de los Angeles Coutino-Coutino, M., Gonzalez-Pozos, S., Bello-Lopez, J. M., Lopez-Ramirez, M. P., Ramirez-Villanueva, D. A., and Dendooven, L. (2013) Natronobacterium texcoconense sp. nov., a haloalkaliphilic archaeon isolated from soil of a former lake. *Int J Syst Evol Microbiol* **63**, 4163-4166

158. Itoh, T., Yamaguchi, T., Zhou, P., and Takashina, T. (2005) Natronolimnobius baerhuensis gen. nov., sp. nov. and Natronolimnobius innermongolicus sp. nov., novel haloalkaliphilic archaea isolated from soda lakes in Inner Mongolia, China. *Extremophiles* **9**, 111-116

159. Burns, D. G., Janssen, P. H., Itoh, T., Minegishi, H., Usami, R., Kamekura, M., and Dyall-Smith, M. L. (2010) Natronomonas moolapensis sp. nov., non-alkaliphilic isolates recovered from a solar saltern crystallizer pond, and emended description of the genus Natronomonas. *Int J Syst Evol Microbiol* **60**, 1173-1176

160. Gonzalez, O., Oberwinkler, T., Mansueto, L., Pfeiffer, F., Mendoza, E., Zimmer, R., and Oesterhelt, D. (2010) Characterization of growth and metabolism of the haloalkaliphile Natronomonas pharaonis. *PLoS Comput Biol* **6**, e1000799

161. Cui, H. L., Tohty, D., Liu, H. C., Liu, S. J., Oren, A., and Zhou, P. J. (2007) Natronorubrum sulfidifaciens sp. nov., an extremely haloalkaliphilic archaeon isolated from Aiding salt lake in Xin-Jiang, China. *Int J Syst Evol Microbiol* **57**, 738-740

162. Walker, C. B., de la Torre, J. R., Klotz, M. G., Urakawa, H., Pinel, N., Arp, D. J., Brochier-Armanet, C., Chain, P. S., Chan, P. P., Gollabgir, A., Hemp, J., Hugler, M., Karr, E. A., Konneke, M., Shin, M., Lawton, T. J., Lowe, T., Martens-Habbena, W., Sayavedra-Soto, L. A., Lang, D., Sievert, S. M., Rosenzweig, A. C., Manning, G., and Stahl, D. A. (2010) Nitrosopumilus maritimus genome reveals unique mechanisms for nitrification and autotrophy in globally distributed marine crenarchaea. *Proc Natl Acad Sci U S A* **107**, 8818-8823

163. Stieglmeier, M., Klingl, A., Alves, R. J., Rittmann, S. K., Melcher, M., Leisch, N., and Schleper, C. (2014) Nitrososphaera viennensis gen. nov., sp. nov., an aerobic and mesophilic, ammonia-oxidizing archaeon from soil and a member of the archaeal phylum Thaumarchaeota. *Int J Syst Evol Microbiol* **64**, 2738-2752

164. Zeng, X., Zhang, X., Jiang, L., Alain, K., Jebbar, M., and Shao, Z. (2013) Palaeococcus pacificus sp. nov., an archaeon from deep-sea hydrothermal sediment. *Int J Syst Evol Microbiol* **63**, 2155-2159

165. Futterer, O., Angelov, A., Liesegang, H., Gottschalk, G., Schleper, C., Schepers, B., Dock, C., Antranikian, G., and Liebl, W. (2004) Genome sequence of Picrophilus torridus and its implications for life around pH 0. *Proc Natl Acad Sci U S A* **101**, 9091-9096

166. Volkl, P., Huber, R., Drobner, E., Rachel, R., Burggraf, S., Trincone, A., and Stetter, K. O. (1993) Pyrobaculum aerophilum sp. nov., a novel nitrate-reducing hyperthermophilic archaeum. *Appl Environ Microbiol* **59**, 2918-2926

167. Feinberg, L. F., Srikanth, R., Vachet, R. W., and Holden, J. F. (2008) Constraints on anaerobic respiration in the hyperthermophilic Archaea Pyrobaculum islandicum and Pyrobaculum aerophilum. *Appl Environ Microbiol* **74**, 396-402

168. Kengen, S. W. M. (2017) 'Pyrococcus furiosus, 30 years on'. *Microb Biotechnol* **10**, 1441-1444

169. Kawarabayasi, Y. (2001) Genome of Pyrococcus horikoshii OT3. *Methods Enzymol* **330**, 124-134

170. Zeng, X., Birrien, J. L., Fouquet, Y., Cherkashov, G., Jebbar, M., Querellou, J., Oger, P., Cambon-Bonavita, M. A., Xiao, X., and Prieur, D. (2009) Pyrococcus CH1, an obligate piezophilic hyperthermophile: extending the upper pressure-temperature limits for life. *ISME J* **3**, 873-876

171. Demey, L. M., Miller, C. R., Manzella, M. P., Spurbeck, R. R., Sandhu, S. K., Reguera, G., and Kashefi, K. (2017) The draft genome of the hyperthermophilic archaeon Pyrodictium delaneyi strain hulk, an iron and nitrate reducer, reveals the capacity for sulfate reduction. *Stand Genomic Sci* **12**, 47

172. Anderson, I., Goker, M., Nolan, M., Lucas, S., Hammon, N., Deshpande, S., Cheng, J. F., Tapia, R., Han, C., Goodwin, L., Pitluck, S., Huntemann, M., Liolios, K., Ivanova, N., Pagani, I., Mavromatis, K., Ovchinikova, G., Pati, A., Chen, A., Palaniappan, K., Land, M., Hauser, L., Brambilla, E. M., Huber, H., Yasawong, M., Rohde, M., Spring, S., Abt, B., Sikorski, J., Wirth, R., Detter, J. C., Woyke, T., Bristow, J., Eisen, J. A., Markowitz, V., Hugenholtz, P., Kyrpides, N. C., Klenk, H. P., and Lapidus, A. (2011) Complete genome sequence of the hyperthermophilic chemolithoautotroph Pyrolobus fumarii type strain (1A). *Stand Genomic Sci* **4**, 381-392

173. Dominova, I. N., Sorokin, D. Y., Kublanov, I. V., Patrushev, M. V., and Toshchakov, S. V. (2013) Complete Genome Sequence of Salinarchaeum sp. Strain HArcht-Bsk1T, Isolated from Hypersaline Lake Baskunchak, Russia. *Genome Announc* **1**

174. Anderson, I. J., Sun, H., Lapidus, A., Copeland, A., Glavina Del Rio, T., Tice, H., Dalin, E., Lucas, S., Barry, K., Land, M., Richardson, P., Huber, H., and Kyrpides, N. C. (2009) Complete genome sequence of Staphylothermus marinus Stetter and Fiala 1986 type strain F1. *Stand Genomic Sci* **1**, 183-188

175. Servin-Garciduenas, L. E., and Martinez-Romero, E. (2014) Draft Genome Sequence of the Sulfolobales Archaeon AZ1, Obtained through Metagenomic Analysis of a Mexican Hot Spring. *Genome Announc* **2**

176. Chen, L., Brugger, K., Skovgaard, M., Redder, P., She, Q., Torarinsson, E., Greve, B., Awayez, M., Zibat, A., Klenk, H. P., and Garrett, R. A. (2005) The genome of Sulfolobus acidocaldarius, a model organism of the Crenarchaeota. *J Bacteriol* **187**, 4992-4999

177. She, Q., Singh, R. K., Confalonieri, F., Zivanovic, Y., Allard, G., Awayez, M. J., Chan-Weiher, C. C., Clausen, I. G., Curtis, B. A., De Moors, A., Erauso, G., Fletcher, C., Gordon, P. M., Heikamp-de Jong, I., Jeffries, A. C., Kozera, C. J., Medina, N., Peng, X., Thi-Ngoc, H. P., Redder, P., Schenk, M. E., Theriault, C., Tolstrup, N., Charlebois, R. L., Doolittle, W. F., Duguet, M., Gaasterland, T., Garrett, R. A., Ragan, M. A., Sensen, C. W., and Van der Oost, J. (2001) The complete genome of the crenarchaeon Sulfolobus solfataricus P2. *Proc Natl Acad Sci U S A* **98**, 7835-7840

178. Dai, X., Wang, H., Zhang, Z., Li, K., Zhang, X., Mora-Lopez, M., Jiang, C., Liu, C., Wang, L., Zhu, Y., Hernandez-Ascencio, W., Dong, Z., and Huang, L. (2016) Genome Sequencing of Sulfolobus sp. A20 from Costa Rica and Comparative Analyses of the Putative Pathways of Carbon, Nitrogen, and Sulfur Metabolism in Various Sulfolobus Strains. *Front Microbiol* **7**, 1902

179. Yanai, H., Doi, K., and Ohshima, T. (2009) Sulfolobus tokodaii ST0053 produces a novel thermostable, NAD-dependent medium-chain alcohol dehydrogenase. *Appl Environ Microbiol* **75**, 1758-1763

180. Jung, M. Y., Kim, J. G., Sinninghe Damste, J. S., Rijpstra, W. I., Madsen, E. L., Kim, S. J., Hong, H., Si, O. J., Kerou, M., Schleper, C., and Rhee, S. K. (2016) A hydrophobic ammonia-oxidizing archaeon of the Nitrosocosmicus clade isolated from coal tar-contaminated sediment. *Environ Microbiol Rep* **8**, 983-992

181. Kuwabara, T., Minaba, M., Ogi, N., and Kamekura, M. (2007) Thermococcus celericrescens sp. nov., a fast-growing and cell-fusing hyperthermophilic archaeon from a deep-sea hydrothermal vent. *Int J Syst Evol Microbiol* **57**, 437-443

182. Huber, R., Stöhr, J., Hohenhaus, S., Rachel, R., Burggraf, S., Jannasch, H. W., and Stetter, K. O. (1995) Thermococcus chitonophagus sp. nov., a novel, chitin-degrading, hyperthermophilic archaeum from a deep-sea hydrothermal vent environment. *Archives of Microbiology* **164**, 10

183. Jolivet, E., L'Haridon, S., Corre, E., Forterre, P., and Prieur, D. (2003) Thermococcus gammatolerans sp. nov., a hyperthermophilic archaeon from a deep-sea hydrothermal vent that resists ionizing radiation. *Int J Syst Evol Microbiol* **53**, 847-851

184. Fukui, T., Atomi, H., Kanai, T., Matsumi, R., Fujiwara, S., and Imanaka, T. (2005) Complete genome sequence of the hyperthermophilic archaeon Thermococcus kodakaraensis KOD1 and comparison with Pyrococcus genomes. *Genome Res* **15**, 352-363

185. Lee, H. S., Kang, S. G., Bae, S. S., Lim, J. K., Cho, Y., Kim, Y. J., Jeon, J. H., Cha, S. S., Kwon, K. K., Kim, H. T., Park, C. J., Lee, H. W., Kim, S. I., Chun, J., Colwell, R. R., Kim, S. J., and Lee, J. H. (2008) The complete genome sequence of Thermococcus onnurineus NA1 reveals a mixed heterotrophic and carboxydotrophic metabolism. *J Bacteriol* **190**, 7491-7499

186. Miroshnichenko, M. L., Gongadze, G. M., Rainey, F. A., Kostyukova, A. S., Lysenko, A. M., Chernyh, N. A., and Bonch-Osmolovskaya, E. A. (1998) Thermococcus gorgonarius sp. nov. and Thermococcus pacificus sp. nov.: heterotrophic extremely thermophilic archaea from New Zealand submarine hot vents. *Int J Syst Bacteriol* **48 Pt 1**, 23-29

187. Miroshnichenko, M. L., Hippe, H., Stackebrandt, E., Kostrikina, N. A., Chernyh, N. A., Jeanthon, C., Nazina, T. N., Belyaev, S. S., and Bonch-Osmolovskaya, E. A. (2001) Isolation and characterization of Thermococcus sibiricus sp. nov. from a Western Siberia high-temperature oil reservoir. *Extremophiles* **5**, 85-91

188. Jung, J. H., Kim, Y. T., Jeon, E. J., Seo, D. H., Hensley, S. A., Holden, J. F., Lee, J. H., and Park, C. S. (2014) Complete genome sequence of hyperthermophilic archaeon Thermococcus sp. ES1. *J Biotechnol* **174**, 14-15

189. Toshchakov, S. V., Korzhenkov, A. A., Samarov, N. I., Mazunin, I. O., Mozhey, O. I., Shmyr, I. S., Derbikova, K. S., Taranov, E. A., Dominova, I. N., Bonch-Osmolovskaya, E. A., Patrushev, M. V., Podosokorskaya, O. A., and Kublanov, I. V. (2015) Complete genome sequence of and proposal of Thermofilum uzonense sp. nov. a novel hyperthermophilic crenarchaeon and emended description of the genus Thermofilum. *Stand Genomic Sci* **10**, 122

190. Mardanov, A. V., Kochetkova, T. V., Beletsky, A. V., Bonch-Osmolovskaya, E. A., Ravin, N. V., and Skryabin, K. G. (2012) Complete genome sequence of the hyperthermophilic cellulolytic crenarchaeon "Thermogladius cellulolyticus" 1633. *J Bacteriol* **194**, 4446-4447

191. Siebers, B., Zaparty, M., Raddatz, G., Tjaden, B., Albers, S. V., Bell, S. D., Blombach, F., Kletzin, A., Kyrpides, N., Lanz, C., Plagens, A., Rampp, M., Rosinus, A., von Jan, M., Makarova, K. S., Klenk, H. P., Schuster, S. C., and Hensel, R. (2011) The complete genome sequence of Thermoproteus tenax: a physiologically versatile member of the Crenarchaeota. *PLoS One* **6**, e24222

192. Mardanov, A. V., Gumerov, V. M., Beletsky, A. V., Prokofeva, M. I., Bonch-Osmolovskaya, E. A., Ravin, N. V., and Skryabin, K. G. (2011) Complete genome sequence of the thermoacidophilic crenarchaeon Thermoproteus uzoniensis 768-20. *J Bacteriol* **193**, 3156-3157

193. Spring, S., Rachel, R., Lapidus, A., Davenport, K., Tice, H., Copeland, A., Cheng, J. F., Lucas, S., Chen, F., Nolan, M., Bruce, D., Goodwin, L., Pitluck, S., Ivanova, N., Mavromatis, K., Ovchinnikova, G., Pati, A., Chen, A., Palaniappan, K., Land, M., Hauser, L., Chang, Y. J., Jeffries, C. C., Brettin, T., Detter, J. C., Tapia, R., Han, C., Heimerl, T., Weikl, F., Brambilla, E., Goker, M., Bristow, J., Eisen, J. A., Markowitz, V., Hugenholtz, P., Kyrpides, N. C., and Klenk, H. P. (2010) Complete genome sequence of Thermosphaera aggregans type strain (M11TL). *Stand Genomic Sci* **2**, 245-259

194. Itoh, T., Suzuki, K., and Nakase, T. (2002) Vulcanisaeta distributa gen. nov., sp. nov., and Vulcanisaeta souniana sp. nov., novel hyperthermophilic, rod-shaped crenarchaeotes isolated from hot springs in Japan. *Int J Syst Evol Microbiol* **52**, 1097-1104
